# Monocyte-derived cells invade brain parenchyma and amyloid plaques in human Alzheimer’s disease hippocampus

**DOI:** 10.1186/s40478-023-01530-z

**Published:** 2023-02-28

**Authors:** Clara Muñoz-Castro, Marina Mejias-Ortega, Elisabeth Sanchez-Mejias, Victoria Navarro, Laura Trujillo-Estrada, Sebastian Jimenez, Juan Antonio Garcia-Leon, Juan Jose Fernandez-Valenzuela, Maria Virtudes Sanchez-Mico, Carmen Romero-Molina, Ines Moreno-Gonzalez, David Baglietto-Vargas, Marisa Vizuete, Antonia Gutierrez, Javier Vitorica

**Affiliations:** 1 Dpto. Bioquimica Y Biologia Molecular, Facultad de Farmacia, Universidad de Sevilla, C/ Prof. Garcia Gonzalez 2, 41012 Seville, Spain; 2 Instituto de Biomedicina de Sevilla (IBiS)-Hospital Universitario Virgen del Rocío/CSIC/Universidad de Sevilla, 41013 Seville, Spain; 3 Dpto. Biologia Celular, Genetica y Fisiologia, Instituto de Investigación Biomedica de Malaga-IBIMA, Facultad de Ciencias, Universidad de Malaga, Campus de Teatinos S/N, 29071 Malaga, Spain; 4 Centro de Investigacion Biomedica en Red Sobre Enfermedades Neurodegenerativas (CIBERNED), 28031 Madrid, Spain

**Keywords:** Alzheimer’s disease, Myeloid cells, Microglia, Brain infiltration, Human hippocampus, Amyloid plaques

## Abstract

Microglia are brain-resident myeloid cells and play a major role in the innate immune responses of the CNS and the pathogenesis of Alzheimer's disease (AD). However, the contribution of nonparenchymal or brain-infiltrated myeloid cells to disease progression remains to be demonstrated. Here, we show that monocyte-derived cells (MDC) invade brain parenchyma in advanced stages of AD continuum using transcriptional analysis and immunohistochemical characterization in post-mortem human hippocampus. Our findings demonstrated that a high proportion (60%) of demented Braak V–VI individuals was associated with up-regulation of genes rarely expressed by microglial cells and abundant in monocytes, among which stands the membrane-bound scavenger receptor for haptoglobin/hemoglobin complexes or Cd163. These Cd163-positive MDC invaded the hippocampal parenchyma, acquired a microglial-like morphology, and were located in close proximity to blood vessels. Moreover, and most interesting, these invading monocytes infiltrated the nearby amyloid plaques contributing to plaque-associated myeloid cell heterogeneity. However, in aged-matched control individuals with hippocampal amyloid pathology, no signs of MDC brain infiltration or plaque invasion were found. The previously reported microglial degeneration/dysfunction in AD hippocampus could be a key pathological factor inducing MDC recruitment. Our data suggest a clear association between MDC infiltration and endothelial activation which in turn may contribute to damage of the blood brain barrier integrity. The recruitment of monocytes could be a consequence rather than the cause of the severity of the disease. Whether monocyte infiltration is beneficial or detrimental to AD pathology remains to be fully elucidated. These findings open the opportunity to design targeted therapies, not only for microglia but also for the peripheral immune cell population to modulate amyloid pathology and provide a better understanding of the immunological mechanisms underlying the progression of AD.

**Supplementary Information:**

The online version contains supplementary material available at 10.1186/s40478-023-01530-z.

## Introduction

Alzheimer’s disease (AD) is a life-threatening neurodegenerative condition that progressively impairs cognitive function and daily living skills. It is the most common pathologic cause of dementia in the late adult life, and no cure is currently available. AD is neuropathologically characterized by abnormal aggregation of the amyloid-β (Abeta) peptides in plaques and hyperphosphorylated neuronal tau protein in neurofibrillary tangles, synaptic damage, and neuronal loss, together with glial-mediated innate immune responses [14].

In the last years, genome-wide association studies (GWAS) have identified many AD-risk genes related to microglial function and brain innate immunity [8]. Microglia, the brain parenchymal-resident myeloid cells and main immune cell components of the central nervous system (CNS), play a key role in brain homeostasis and AD pathogenesis [35, 43]. Under physiological conditions, other cells of the myeloid linage, such as macrophages associated with the CNS border, localized in the choroid plexus, leptomeningeal and perivascular spaces, as well as circulating monocytes and lymphocytes, among others, constantly surveil the interface between blood or cerebrospinal fluid and brain parenchyma [6, 18, 26]. Indeed, inflammatory neurological diseases are frequently associated with disruption of the blood–brain barrier (BBB) and peripheral immune cell infiltration [32]. Typical examples are infiltration of lymphocytes and monocyte-derived cells (MDC) in multiple sclerosis and amyotrophic lateral sclerosis, which seem to play an important role in the development of these neurodegenerative conditions [3, 26, 42, 66].

In post-mortem brain tissue from individuals with AD, as well as AD transgenic mice, CD4+ and CD8+ T lymphocytes are present in both vascular endothelium and brain parenchyma [13, 32, 57]. However, to date it is unclear whether circulating monocytes can infiltrate AD brains and differentiate into microglial cells [42], due to the difficulty in differentiating brain-resident microglia from infiltrating myeloid populations as they show similar morphology and express mostly the same markers [4].

The microglial population self-renews autonomously without the contribution of bone marrow-derived peripheral myeloid cells under steady state conditions [49]. However, microglial dysfunction, including impaired repopulation capacity, as occurs in AD brains [35, 51], could trigger monocyte recruitment to populate brain tissue. In fact, microglial depletion strategies induced a rapid repopulation by peripherally-derived macrophages [9, 21, 31, 41, 44, 58]. The presence of peripheral-derived monocytes in AD brain parenchyma and assessing their contribution to AD pathology remain a topic of debate.

In this study, using transcriptional analysis together with immunohistochemical characterization, we provided evidence of circulating monocyte infiltration into the brains of AD. Our results revealed a myeloid subpopulation cluster associated with monocyte-specific genes, but not with the classical microglial signature, in the hippocampus of Braak V–VI individuals. These cells were identified in brain parenchyma tissue by their distinct expression of the Cd163 marker, showed a microglial-like morphology, were located close to blood vessels, and invaded amyloid plaques. However, in aged-matched high pathology controls no parenchymal MDC were detected and plaques were devoid of Cd163-cells. We propose that MDC infiltration was associated with endothelial activation and AD pathology severity. The therapeutic value of targeting brain-resident myeloid cells and peripheral monocytes to treat AD needs to be explored.

## Materials and methods

### Human brain samples

Medial temporal lobe (hippocampal and parahippocampal regions) samples were obtained from the tissue bank for neurological research Fundación CIEN (BT-CIEN; Centro de Investigación de Enfermedades Neurologicas; Madrid, Spain), the Neurological Tissue Bank of IDIBELL-Hospital of Bellvitge (Barcelona, Spain), and the Neurological Tissue Bank BioBanco-Hospital clínico-IDIBAPS (Barcelona, Spain). This study was approved by the Portal de Ética de la Investigación Biomédica de Andalucía (PEIBA) de la Consejería de Salud from Andalucía (Spain), as well as by the corresponding biobank ethics committees and by the “Comite de Etica de la Investigacion (CEI), Hospital Virgen del Rocio,” Seville, Spain. Subjects were classified according to Braak stage**.** Demographic and pathological characteristics of these cases are described in Table 1. Only Braak V–VI individuals (77.98 ± 12.48 years) met the clinical and neuropathological criteria for AD. Braak II subjects (77.50 ± 8.81) are considered as non-demented age-matched controls in this study. Younger subjects (49.50 ± 5.95 years) without any known psychiatric or neurological disease, and lack of neuropathological lesions, are referred as Braak 0.

**Table 1:** Summary of the neuropathological samples used in this study

Braak stage	Age (years)	Gender (%)		APOE genotype (%)				Postmortem delay (h)
		Male	Female	ε2ε3	ε3ε3	ε3ε4	ε4ε4	
Unfixed frozen sampes								
Braak 0 (n = 8)	49.50 ± 5.95	62.50	37.50	0.00	66.67	0.00	33.33	7.19 ± 3.29
Braak II (n = 26)	77.50 ± 8.81	53.85	46.15	9.09	77.27	9.09	4.55	8.33 ± 5.05
Braak III–IV (n = 15)	79.42 ± 12.37	46.66	53.33	7.69	61.54	30.77	0.00	5.42 ± 4.80
Braak V–VI (n = 44)	77.98 ± 12.48	43.18	56.82	5.56	69.44	22.22	2.78	8.99 ± 4.55
Fixed samples								
Braak 0 (n = 1)	58	100.00	0.00	–	–	–	––	5.00
Braak II (n = 8)	80.86 ± 6.77	28.57	71.43	–	–	–	–	10.67 ± 5.46
Braak V–VI (n = 14)	72.93 ± 14.06	64.29	35.71	14.29	28.57	57.14	0.00	7.65 ± 3.14

For biochemical characterization, unfixed −80 °C frozen samples were used. For morphological studies, human samples were fixed in cold 4% paraformaldehyde in 0.1 M phosphate buffer (PB) for 24–48 h, cryoprotected in sucrose, stored at −80 °C, sectioned at 30 μm thickness on a freezing microtome, and serially collected in wells containing 0.1 M phosphate buffer saline (PBS) and 0.02% sodium azide. The anatomical boundaries of the hippocampal and parahippocampal regions were identified in Nissl-stained sections by their cytoarchitecture and location using the human brain atlas [16]. The areas located between the stereotaxic coordinates of Bregma 9.3 mm and 34.6 mm were analyzed.

### RNA, DNA and protein extraction

Total RNA, DNA and proteins were extracted using TRIsure isolation reagent (Bioline) following the manufacturer recommendations. RNA was purified using the RNeasy Mini Kit (Quiagen) and RNA integrity (RIN; 5.66 ± 1.06) was determined using RNA Nano 6000 (Agilent). RNA and DNA were quantified using a NanoDrop 2000 spectrophotometer (Thermo Fischer). Retrotranscription (RT) was performed with the High Capacity cDNA Reverse Transcription Kit (Applied Biosystem) using 4 μg of total RNA as template. Proteins were quantified using the Lowry method.

### Quantitative real-time RT-PCR

The real-time RT-PCR reaction mixture was performed in a final volume of 20 μl containing 40 ng of cDNA, iTaq Universal Probes Supermix (Bio-Rad) (2X), and Taqman Gene Expression Assay (Applied Biosystem; Additional file 1: Table S1) (20X). Amplification and measurement were performed by ABI PRISM Sequence Detection Systems 7900 (Applied Biosystems). The reaction was first incubated at 50° C for 2 min, 95° C for 10 min, and then 40 cycles at 95° C for 15 s and 60° C for 1 min. For the quantification of cDNA level, we used the comparative double delta cycle threshold (Ct) method (2^−ΔΔCt^) [30, 46], using GAPDH as the housekeeping gene and Braak II subjects as the reference group. The Ct values were calculated with software provided by Applied Biosystems (SDS 1.7). To validate GAPDH as a housekeeper gene, cDNA levels were also determined by β-actin, 18S, UBE2D2, CYC1, and RPL13. We observed a very significant linear correlation (*p* < 0.0001) among Ct of GAPDH and any other housekeeper genes (β-actin, r = 0.863; 18S, r = 0.565; UBE2D2, r = 0.880; CYC1, r = 0.945; and RPL13, r = 0.834). Thus, normalization using any of them produced identical results.

### Array microfluidic cards

Taqman Array Microfluidic Cards (Thermo Fisher) were custom-made with the probes described in Additional file 1: Table S2 and the manufacturer's protocol was followed. Briefly, we loaded 100 μL of each sample-specific PCR reaction mix, composed of Taqman mastermix (Thermo Scientific) (2x), and cDNA at 10 ng/μl. Eight samples per card, balanced for Braak stage, were run simultaneously. We vertically centrifuged the plate at 1200 rpm for 2 min on a Sorvall ST40R centrifuge (Thermo Scientific). After sealing, the plate was read in the ViiATM 7 Real-Time PCR System (Applied Biosystems). Similarly to real-time RT-PCR, the Ct comparison method was used to quantify cDNA levels. In this case, the amount of cDNA was normalized by the geometric mean of the four housekeeping genes (see Additional file 1: Table S2). The expression of the different genes was referred to the average expression of the Braak II group (n = 16 different individuals).

### Hierarchical clustering and gene set score

Clustering analysis was performed in an unsupervised manner, using the public domain programming environment and language R (version 3.6.0). After Z-score normalization, we confirmed an inherent cluster structure and genes were used to generate a heatmap and perform hierarchical clustering to classify genes or subjects with a similar profile. Clustering was performed with Ward´s linkage method using Manhattan distance.

*The gene set score* was used to quantitatively calculate variations in expression of different markers, associated with the same cell type [15]. Concisely, the gene set score was calculated for each sample 'j' using the value 'eij', which is the normalized expression level of gene 'i' in sample 'j'. We calculate the center gene expression matrix "c_ij_" with the following equation: ${c}_{ij}={e}_{ij}-\frac{1}{{n}_{s}}{\Sigma }_{j}{e}_{ij}$; where "n_s_" is the number of samples. The gene set score for sample "j" (S_j_) is defined as the average of the matrix “c_ij_”: ${S}_{j}=\frac{1}{{n}_{g}}{\Sigma }_{i}{c}_{ij}$; where "n_g_" is the number of genes which constitutes the cluster.

### Apolipoprotein E (APOE) genotyping

For determining the APOE epsilon alleles ε2, ε3, and ε4, manufacturer protocol from Applied Biosystems TaqMan SNP Genotyping Assays C___3084793 and C____904973 (Thermo Fisher Scientific) was followed. C___3084793 and C____904973 identify the single-nucleotide polymorphisms (SNP) rs429358 and rs7412, respectively. Briefly, the T allele at rs429358 and the C allele at rs7412 indicate the ɛ3 allele, whereas the T allele at both SNPs identify the ɛ2 allele, and the C allele at both positions determine the ɛ4 allele. PCR amplification reactions were performed in a final 10 µl volume containing 100 ng genomic DNA, TaqmanTM Genotyping Master Mix (2X), and TaqmanTM SNP Genotyping Assay (50X). The reaction was incubated at 50 $^\circ$C for 2 min, followed by 95 °C for 10 min, and 40 cycles at 95 °C for 15 s and 64 °C (rs429358) or 61 °C (rs7412) for 1 min. The fluorescent signal generated by PCR amplification is detected by ABI PRISM Sequence Detection Systems 7900 (Applied Biosystems) and the allelic discrimination analysis is performed by ABI Prism 7900 SDS Software (Applied Biosystems).

### Immunostaining

First, for general antigen retrieval, free-floating sections were heated at 80 °C for 20 min in 50 mM citrate buffer pH 6.0 and then, sections were treated with 3% hydrogen peroxide/ 10% methanol in PBS pH 7.4 for 20 min to inhibit endogenous peroxidase. Subsequently, nonspecific staining was avoided using 5% goat or horse serum (Sigma-Aldrich) in PBS. For single labeling light microscopy, sections were incubated with the primary antibody (anti-Iba1 or anti-Cd163, see antibodies list) for 24–72 h at room temperature followed by the corresponding biotinylated secondary antibody for 1 h at room temperature (1:500 dilution, Vector Laboratories). Subsequently, incubation with horseradish peroxidase conjugated with streptavidin was performed for 90 min (1:2000 dilution, Sigma-Aldrich) was performed. Finally, the peroxidase reaction was visualized with 0.05% of 3–3′-diaminobenzidine tetrahydrochloride (DAB, Sigma-Aldrich), 0.03% of nickel ammonium sulfate and 0.01% of hydrogen peroxide in PBS. For double immunoperoxidase staining (Iba1/Abeta, Cd163/Abeta, Cd45/Abeta, Cd163/laminin) the first peroxidase reaction was carried out with DAB solution containing nickel ammonium sulfate to get a dark blue end product while the second peroxidase reaction was developed with DAB only, obtaining a brown reaction end product. The sections of the control and AD brains were simultaneously assayed using the same batches of solutions to minimize variability in immunolabeling conditions and the specificity of the immune reactions was controlled by omitting primary antisera.

For double and triple immunofluorescence labeling (Mrc1/Cd163, Cd163/Iba1/Abeta, Cd163/Tmem119/Abeta or Iba1/Trem2/Cd163), sections were incubated sequentially with primary antibodies followed by the corresponding Alexa 405/488/568/Cy5™ secondary antibodies (1:1000 dilution, Invitrogen) and DAPI (Sigma–Aldrich; 1:1,000) staining. Z-stacks images were taken with a Leica TCS SP8 confocal microscope and 3D-analyzed with IMARIS software (version 9.5.1, Bitplane Inc.)

### Primary antibodies list

The following primary antibodies were used for immunolabeling experiments: anti-oligomeric/fibrillar Abeta rabbit polyclonal OC (1:5000, Merck Millipore); anti-Iba1 rabbit polyclonal (1:1000, Wako); anti-Iba1 goat polyclonal (1:1000, Abcam); anti-Cd45 rabbit polyclonal (1:1000 dilution, Abcam); anti-Cd163 mouse monoclonal (1:500, Novocastra); anti-Trem2 rabbit polyclonal (1:200, LS BIO); anti-Mrc1 rabbit polyclonal (1:1000, Sigma-Aldrich); anti-laminin rabbit polyclonal (1:500, Sigma-Aldrich); anti-Tmem119 rabbit polyclonal (1:500, Sigma-Aldrich).

### Quantitative image analyses

#### Myeloid cell loading

Was defined as the percentage of total hippocampal area immunolabeled for Iba1 or Cd163. Whole hippocampal sections from non-demented controls (Braak II; n = 8; 2 sections/ individual) and AD cases (Braak V–VI; n = 11–14; 2 sections/individual) stained with Iba1 or Cd163 were digitalized using a VS120 high-throughput virtual slide system (Olympus, Denmark) connected to Olympus BX61VS microscope with a high-resolution digital color camera (VC50 Olympus). Sections were scanned with 40 × objective (NA = 0.9) with a resolution of 0.175 µm/pixel (VSI file format). A focus map, created by selecting multiple coordinates with the optimal Z position, was automatically generated across the entirety section using a software-based autofocus function in the color brightfield mode. Quality parameters were not modified at any step staying uniform in all samples: gain (white balance) R:1.87; G1.00; B1.78; brightness 0.00, contrast 1.00, gamma 1.00, sharpness 6.00, and saturation 0.00)The high quality virtual slides were viewed using the Olyvia 2.6 software (Olympus, Denmark) and images from the region of interest were extracted (10–15 images/section; image size of 1879 × 872 pixels; pixel size = 72ppp). Quantitative analyses were performed using the Visilog 6.3 image analysis software (Noesis, France). Thus, the 24-bit color digital images were calibrated (scale bar of 200 μm = 117 pixels; 0.585 pixels/μm) and converted to 8-bit gray scale images. After delimiting the reference area (excluding tissue ruptures, large blood vessels or white matter areas), images were binarized using a threshold level mask that was fixed between a range of 170–180 of intensity and manually set for each image to ensure a reliable quantification. The Iba1-positive o CD163-positive coverage area was calculated as sum labeled area measured/sum total area analyzed) × 100. All slides sampled were taken over for the sums, and a single burden was computed for each individual. The mean and standard deviation (SD) of the corresponding loadings were determined using all available data. To make accurate comparison between study groups tissue samples and sections were simultaneously processed using standardized handling and immunostaining protocols.

#### Spatial distribution of myeloid cells around vessels

Whole-slide imaging of hippocampal sections from AD brains (n = 9 Braak V–VI individuals), which were double immunostained with Cd163 or Iba1 and OC antibodies, was acquired at 40 × magnification using the automated virtual microscopy system (Olympus VS120, Japan) as described above. Images were further operated on ImageJ 1.52p image analysis system (NIH, Behtesda, Maryland, USA). Blood vessels (n = 63) were manually outlined, and three concentric circles of fixed radius (85 μm) were delineated surrounding each vessel. Then, the area covered by CD163-positive cells, Iba1-positive cells or OC-positive amyloid plaques was quantified in the ring-shaped regions bounded by the three concentric circles for each image, after applying a threshold level mask (a range of 170–180 of intensity values for Cd163 and Iba1, and 150–160 for OC, was used).

#### Periplaque myeloid cell quantification

High resolution confocal Z-stack images from AD hippocampal sections (n = 6 Braak V–VI individuals; 2 sections per case; 73 total plaques analyzed) with triple (Cd163/Iba1/Trem2) immunofluorescence staining were taken using a Leica TCS SP8 confocal microscopy (Leica Microsystems CMS GmbH,Wetzlar) with a 63X oil immersion objectiv lens. The number of periplaque myeloid cells single immunolabeled for Iba1, Cd163 or Trem2, as well double labeled for Iba1/Cd163 or Trem2/Cd163, was counted using ImageJ software. Only cells with a clearly visible cell body (DAPI-positive) were quantified. To assess the colocalization of Iba1 and Cd163, as well as Trem2 and Cd163, single planes from the confocal image stacks were split into individual channels.

### Western Blot

Western blots were performed as previously described [11, 51]. In brief, to detect monomeric Abeta a 16% SDS-Tris-Tricine-PAGE was performed. Proteins were transferred to PVDF membranes (Immobilon-P Transfer Membrane, Merck-Millipore) and incubated with mouse monoclonal anti-Abeta-N-terminal antibody (82E1, 1:2000, IBL) overnight at 4° C. For total and phosphorylated Tau, we performed a 4% to 20% SDS-Tris–Glycine-PAGE (Bio-Rad) and transferred to nitrocellulose membranes (Hybond-C extra, Amersham). Mouse monoclonal anti-Tau (Tau46, 1:1000, Cell Signaling), mouse monoclonal anti-phosphoTau^Ser202, Thr205^ (AT8, 1:1000, Thermo Scientific), and mouse monoclonal anti-phosphoTau^Thr212, Ser214^ (AT100, Innogenetics, 1:1000) were used. The membranes were then incubated with a goat anti-mouse IgG, horseradish peroxidase (HRP)-linked antibody (1:10,000, Cell Signaling). Proteins were visualized using Pierce ECL 2 Western Blotting Substrate (Thermo Scientific) in the ChemiDocTM Touch Imaging System (Bio-rad) and quantified using Image Lab software (Bio-Rad). For normalization purposes, proteins were first estimated by Lowry and protein loading was corrected by GAPDH (rabbit monoclonal anti-GAPDH antibody; 14C10 1:10,000, Cell Signaling). The phospho-Tau proteins were referred to total Tau levels.

### Statistical analysis

Statistical analysis was performed using GraphPad Prism 8 (Prism) or SPSS software (IBM® SPSS® Statistics, v25). The normality of the data was evaluated using the Kolmogorov–Smirnov test. Nonnormally distributed data were represented as individual dots or using violin plots with the median and interquartile range and individual subject values. Data were compared using the Mann–Whitney U test (for two groups comparisons), Kruskal–Wallis tests (more than two groups) or Friedman test (for repeated measures) both followed by Dunn's post hoc test. Linear correlations were analyzed using Spearman correlation. The Chi-square test or Fisher's exact test was used to analyze the relation between qualitative variables. The significance was established at 95% confidence.

## Results

### Transcriptional characterization of a specific myeloid CD163 cluster in the hippocampus of AD brains

**Fig. 1:**
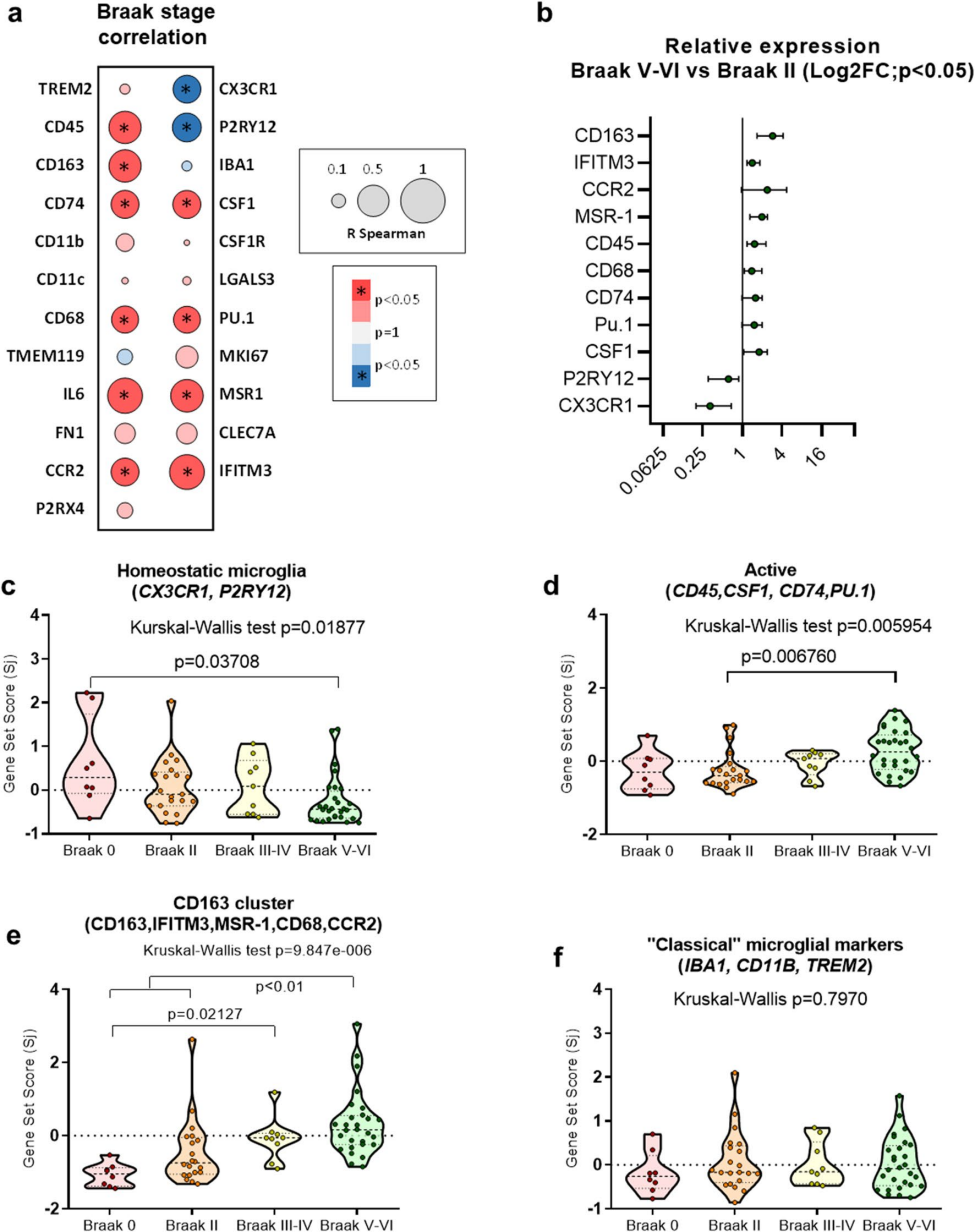
Expression analysis of microglial/myeloid genes in human post-mortem hippocampal samples from Braak 0 to Braak V–VI (AD cases). The expression of the different microglial/myeloid genes was tested in human hippocampal samples classified by Braak stages, from Braak 0 (no pathology) to Braak VI (see Additional file 1: Fig. S1 for individual data). Braak stage dependent changes in expression were first analyzed using Spearman correlation analysis (**a**) and by Braak II vs Braak V–VI direct comparison using Mann Whitney test (**b**). **c** to **e** represented the Braak stage-dependent variation of the gene set score of the three different clusters identified using data shown in **b** (Additional file 1: Fig. S1): homeostatic microglia (**c**), active microglia (**d**) and Cd163 cluster (**e**). (**f**) Expression of “classic” microglial markers (*IBA1*, *CD11B* and *TREM2*). The data were shown as violin plots including the individual cases. Significance, indicated in the figure, was tested using the Kruskal–Wallis test followed by the Dunn test

We first analyzed, using Spearman regression analysis, the possible modifications in the expression of microglial/MDC genes in human hippocampal samples, classified by Braak stage (from Braak 0 to Braak VI, Fig. 1a and Additional file 1: Fig. S1a). Our data showed a highly heterogeneous response. In fact, we did observe a significant positive (increase), negative (decrease), and not significant correlation with the progression of the pathology (Fig. 1a). Furthermore, compared directly with the age-matched non-demented Braak II samples, only a limited number of genes showed significant modifications in the Braak V–VI stage (Mann–Whitney test, *p* < 0.05) (Fig. 1b).

Next, we performed a hierarchical cluster analysis (Additional file 1: Fig. S1b) of genes that change significantly in Braak V–VI samples (Fig. 1b) and identified three main myeloid clusters; homeostatic microglia (hMG) (P2RY12, CX3CR1), activated microglia (aMG) (*PU.1, CSF1, CD45, CD74*) [15, 24, 35, 50]; and a third group including non-classic microglial markers [5, 12] which we termed Cd163 cluster (*CD163, IFITM3, CCR2, CD68, and MSR1*). Next, using the gene set score (Sj) [15], we analyzed whether the gene expression of these clusters was affected by the progression of AD. As shown in Fig. 1c, a significant down-regulation in the expression of hMG genes was produced in the Braak V–VI group together with limited and heterogeneous aMG signature genes (Kruskal–Wallis, *p* = 0.006, Dunn test *p* = 0.006760 in Braak V–VI compared to Braak II, Fig. 1d). These data were consistent with the weak microglial response in the human hippocampus previously described by our group [35, 51]. In fact, the expression of “classic” microglial markers (such as *IBA1, CD11B* and *TREM2*) (Fig. 1f) did not show significant changes in Braak V–VI subjects. In contrast, we observed a clear and highly significant up-regulation of the Cd163 cluster (Fig. 1e, Kruskal–Wallis, *p* = 9.847e−6; post hoc Dunn test, *p* < 0.01, Braak V–VI compared to Braak 0 and II or Braak III-IV versus Braak 0) in this cohort. Therefore, there was a clear mismatch between the Cd163 cluster genes in the AD group compared to the active or classical microglial markers in the same sample cohort.

### Parenchymal and plaque-associated CD163 cells represent a pathological feature of AD hippocampus

**Fig. 2:**
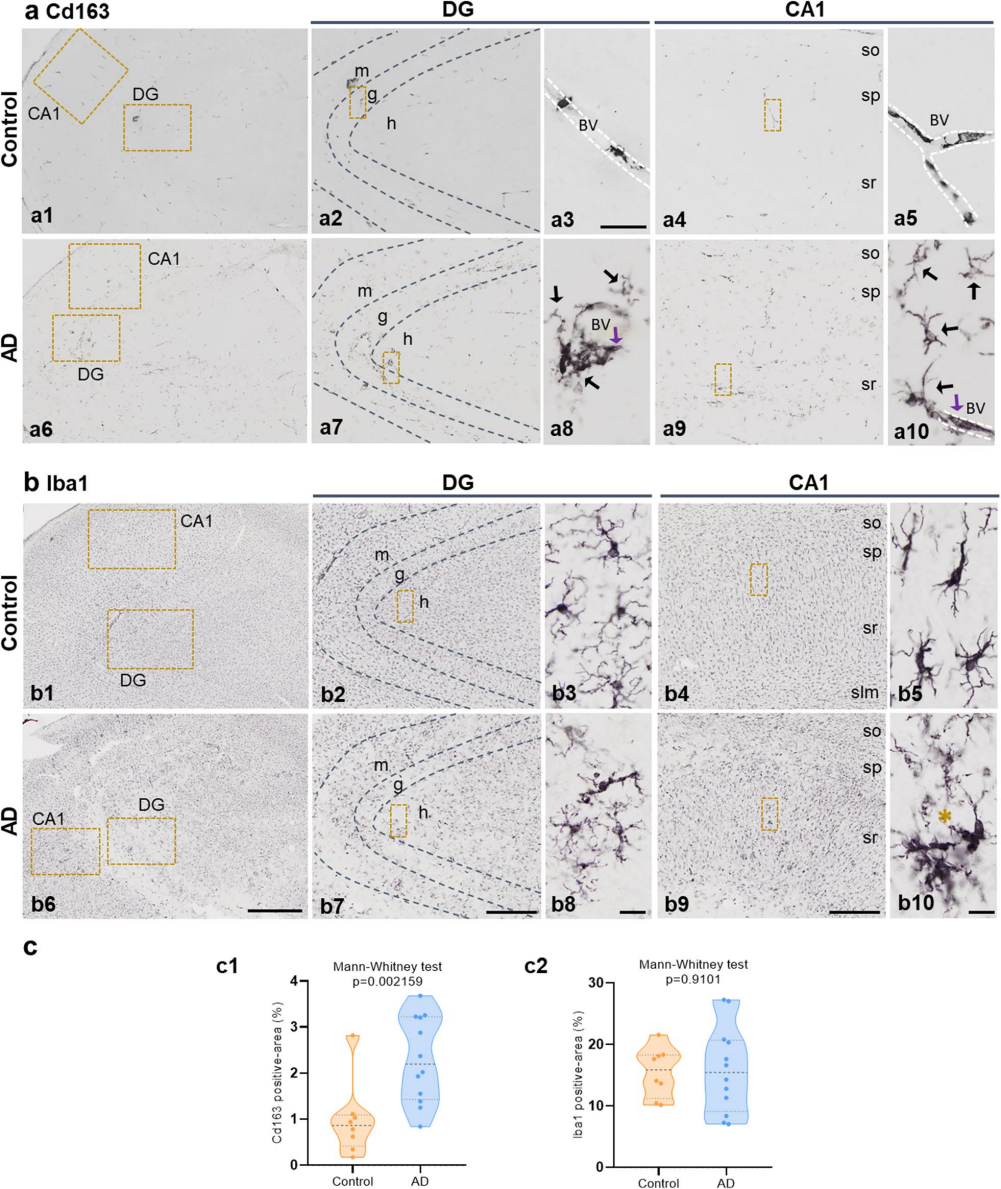
Presence of Cd163-positive cells in the hippocampal parenchyma of AD hippocampus. **(a)** Immunostaining for CD163 in Braak II (age-matched controls, a1–5) and Braak V–VI (AD patients, a6–10) hippocampus. Cd163-positive cells from Braak II individuals (a1) were limited to blood vessels in both DG (a2, boxed area a3) and CA1 (a4, boxed area a5) areas. AD samples (a6) showed Cd163 cells not only associated with blood vessels (purple arrows in a8 and a10), but also distributed throughout the hippocampal parenchyma (black arrows in a8 and a10). **(b)** Immunolabeling for Iba1 in the hippocampus of control (b1–5) and AD (b6–10) individuals. Iba1-microglial cells from Braak II cases (b1) exhibited a homogenous distribution (b2 and b4, boxed areas b3 and b5) compared to AD hippocampus (b6) which included degeneration (b7, boxed area b8) and clustering (b9, boxed area b10). **(c)** Quantitative analysis of the parenchymal area (percentage) covered by Cd163 (c1) and Iba1 (c2) positive cells in control (n = 8) and AD (n = 12) samples. The results are shown as violin plots including the individual cases (dots). Mann–Whitney U test comparison between control and AD groups. BV: blood vessels; CA1: cornu ammonis; DG: dentate gyrus; g: granular layer; h: hilus; m: molecular layer; so: stratum oriens; sp: stratum pyramidale; sr: stratum radiatum; slm: stratum lacunosum-moleculare.* Asterisk indicates Abeta plaque. Scale bars: a1, a6, b1 and b6, 1 mm; a2, a4, a7, a9, b2, b4, b7 and b9, 500 μm; a3, 50 μm; a5, a8, a10, b3, b5, b8 and b10, 20 μm

Next, we analyzed by immunohistochemistry the existence of Cd163 positive cells in the hippocampus of AD (Braak V–VI) hippocampus, compared to control samples (non-demented individuals of Braak II individuals) samples. In parallel, we also analyzed the microglial cell population using the classical Iba1 labeling. As shown in Fig. 2 (a1–5), in control samples Cd163-positive cells were restricted to blood vessels. In fact, control samples showed a very low relative abundance of Cd163 positive cells compared to Iba1 cells (0.97 ± 0.81% Cd163 load vs 15.50 ± 4.08% Iba1 load, n = 8, Fig. 2c). These data were consistent with the already known expression of CD163 in perivascular macrophages (see below) and with the low expression of CD163 detected by qPCR in these samples (see Fig. 1e). However, in the hippocampus of Braak V–VI subjects (Fig. 2a6–10), we also observed Cd163 positive cells located in blood vessels, but much more relevant and opposed to control samples, we also observed Cd163 positive cells present in the hippocampal parenchyma. These parenchymal Cd163 cells showed a ramified morphology and were unevenly distributed throughout the hippocampus, and in many cases a close association with blood vessels was clearly seen (Fig. 2a8 and 10). Interestingly, the density and distribution pattern of Cd163+ cells was completely different from that observed for Iba1 microglial cells (Fig. 2b). Iba1 cells were much more abundant than Cd163 cells (Fig. 2), and showed an even distribution throughout the parenchyma. This homogeneous location of Iba1 cells was better seen in control cases (Fig. 2b1–5), since in the hippocampus of AD (Fig. 2b6–10) microglia undergo profound changes, including activation and clustering around amyloid plaques, and in some regions as dentate gyrus degeneration with dystrophic morphology and the presence of nude areas of Iba1 cells, as previously reported by our group [51].

We confirmed our observations by quantitatively determining the relative abundance of Cd163 positive cells in hippocampal tissue sections. As shown (Fig. 2c1), there was a clear and highly significant increase (0.97 ± 0.81% vs 2.30 ± 0.94%; Mann Whitney test *p* = 0.002159; n = 8 or n = 12 for Braak II and Braak V–VI samples, respectively) in the parenchymal area covered by Cd163 positive cells in Braak V–VI samples. However, no significant differences were detected in the Iba1 positive area (Fig. 2c2). It should be noted that despite the clear increase in Braak V–VI samples, Cd163 positive cells still constituted a minor population compared to microglial cells (2.30 ± 0.94% vs 15.89 ± 7.03% for Cd163 and Iba1 cells, respectively).

**Fig. 3:**
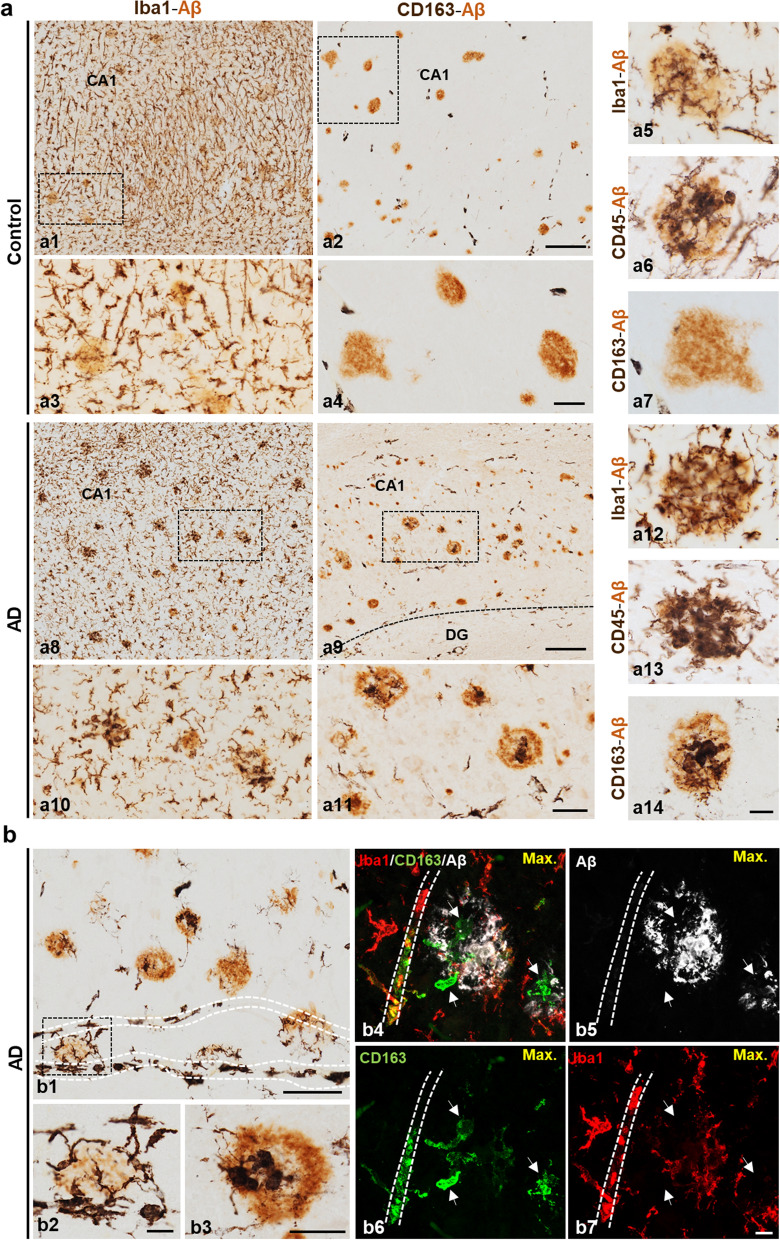
Only Abeta plaques from AD hippocampus are infiltrated with Cd163-positive cells. **(a)** Representative images of Braak II CERAD B (a1–14; control) and Braak V–VI CERAD C (a8–14; AD) cases immunostained for Abeta deposits (OC antibody, light brown) and different myeloid markers (Iba1, Cd163 and Cd45, dark brown). Plaques from the same region of Braak II samples, reactive for microglial markers Iba1 (a1, boxed area a3; higher magnification in a5) and Cd45 (a6), were negative for Cd163 cells (a2, boxed area a4; higher magnification in a7). On the contrary, Abeta deposits from AD hippocampus were infiltrated with Cd163-positive cells (a9, boxed area a11; higher magnification in a14) in addition to Iba1 (a8, boxed area a10) and Cd45 (a13) association. **(b)** Representative images of Braak V–VI samples showing Cd163-infiltrating Abeta plaques (b2–5) in close association with blood vessels (dashed line in b1 and b4-b7). The sections were double immunostained using OC (light brown color) and Cd163 (dark brown color) antibodies (b1–3) for bright field microscopy, or triple immunostained for Abeta (OC, white, b4), Cd163 (green, b5), Iba1 (red, b6) and the combination (b7) for confocal microscopy. CA1: cornu ammonis; DG: dentate gyrus. Scale bars: a1, a2, a8 and a9, 200 μm; b1, 100 μm; a3, a4, a10, a11, b2 and b4, 50 μm; a5, a6, a7, a12, a13, a14, b3 and b5, 20 μm

Next, we studied and compared the association of Cd163-positive cells and microglial Iba1 cells with Abeta plaques in early (Braak II control individuals with amyloid plaques, CERAD B) and advanced (demented Braak V–VI AD cases, CERAD C) stages of the pathology (Fig. 3). As shown (Fig. 3a1–4), despite the presence of relatively abundant Iba1 cells and Abeta plaques, no Cd163 positive cells were observed in the hippocampal parenchyma in Braak II cases. All Cd163 cells were restricted to blood vessels (Fig. 3a2 and a4). Importantly, Iba1 or Cd45 positive and, consequently, activated microglial cells were clearly associated with Abeta plaques (Fig. 3a5, a6). However, no Cd163 cells were associated with these plaques (see Fig. 3a7).

**Fig. 4:**
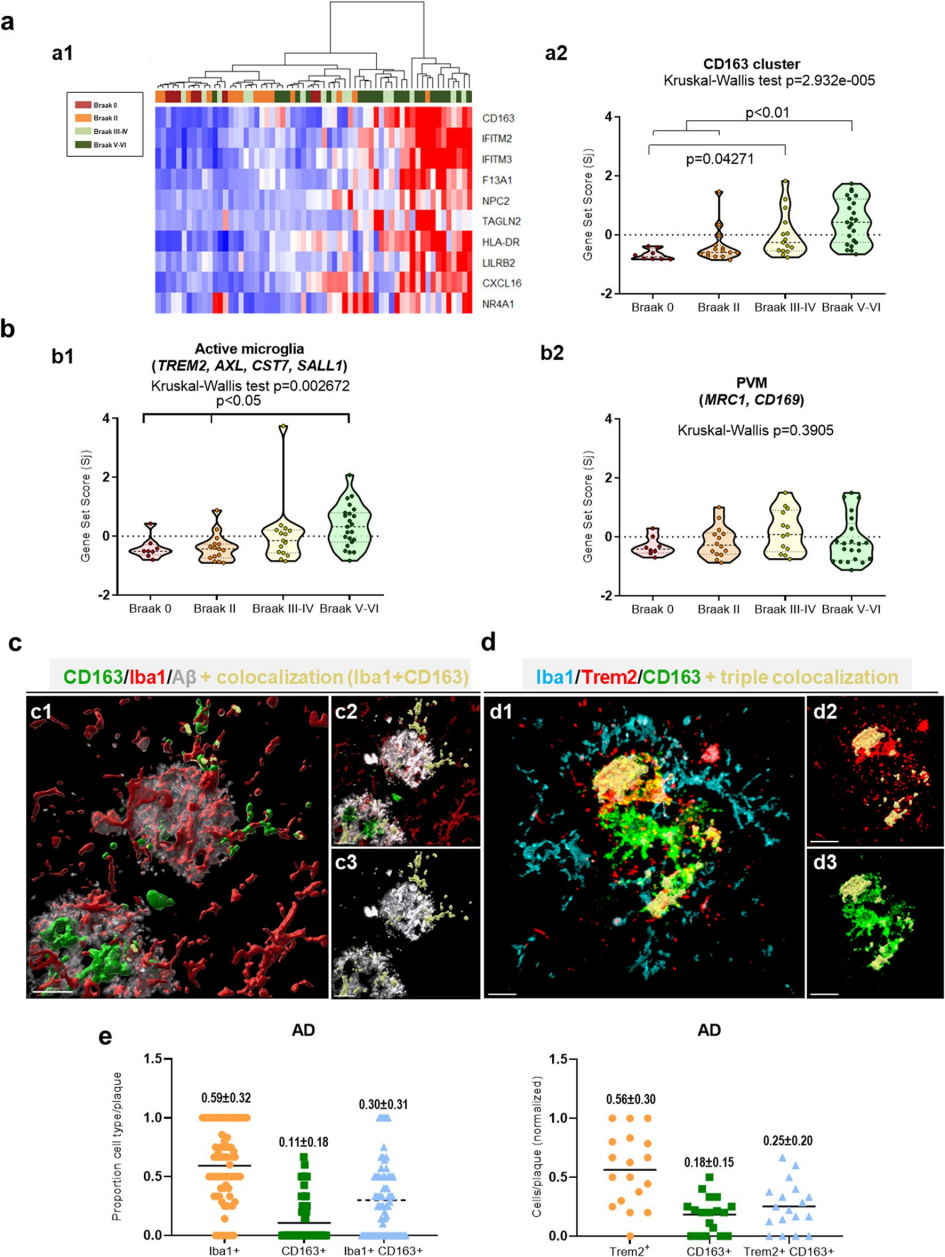
A specific subset of Cd163-cells does not express classical microglial markers. **(a)** Heatmap (a1) and gen-set-score (a2) of the expression of ten selected myeloid derived cell (MDC) specific genes (Cd163-cluster), quantified by Array Microfluidic Cards, in human hippocampus. (a1) Color key code represents Z-score distribution, from −1.5 (blue) to 1.5 (red). Subjects were ordered in an unsupervised manner using hierarchical clustering (Ward´s linkage method, Manhattan distance). At the top of the heatmap, a color bar represents each subject Braak stage (Braak 0, n = 8, Braak II, n = 15; Braak III-IV, n = 15, and Braak V–VI, n = 23). (a2) Braak dependent variations of the gene set score of Cd163-cluster. Note the clear up-regulation of these specific MDC genes mainly in Braak V–VI subjects. **(b)** Graphs represented active microglia (b1) and perivascular macrophages (b2). The expression of all different genes was assayed in parallel using fluidic cards. Data were shown as violin plots including individual samples. Significance, indicated in the figure, was tested using the Kruskal–Wallis test followed by the Dunn test. **(c)** Imaris-generated 3D surfaces images (c1) reveal different plaque-associated myeloid cells in the triple Cd163/Iba1/Abeta immunofluorescence (c2), showing the co-localization (yellow color, c3) between Iba1 and Cd163 in the nearness of two Ab plaques (white color**). (d)** Confocal microscopy of triple Iba1/Trem2/Cd163 immunofluorescence (d1), showing the corresponding Imaris-generated 3D reconstruction of the co-localization channel (yellow color) between the three markers analyzed (d2, d3). **(e)** Graphs showed the quantitative analysis of the different microglial/myeloid cells per Abeta plaque observed in c and d. Each point represented the proportion of the different cell population vs the total number of cells per plaque in AD cases (n = 3–7 BraakV–VI samples and 18–73 plaques for Iba1/Cd163/ or Trem2/Cd163 experiments, respectively). *Scale bars: c1, c2 and c3, 30 μm; d1, d2 and d3, 10 μm*

On the contrary, the presence of Cd163 cells was notable within Abeta plaques in the AD hippocampus (Fig. 3a9, a11 and a14). These plaques also contained activated microglial cells with Iba1 (Fig. 3a8, a10, and a12) and Cd45 (Fig. 3a13) activated microglial cells. In fact, the presence of Cd163 positive cells was observed in 65.43 ± 14.84% (n = 7 different Braak V–VI individuals) of the Abeta plaques in this cohort.

Together, these data demonstrated the presence of Cd163 positive cells in Braak V–VI plaques and also indicated that these cells were not a subset of microglia. In this sense, our immunohistochemical analysis pointed to a peripheral origin of plaque-associated Cd163 cells. As shown in Fig. 3b, Cd163-cells were preferentially located in blood vessels (perivascular macrophages), in the parenchyma nearby these vessels showing a ramified microglial-like morphology, as well as surrounding and infiltrating amyloid plaques located in the proximity of these vessels. Double Iba1/Cd163 immunofluorescence labeling showed that these Cd163-cells were mainly negative for Iba1 and were found in the brain parenchyma in close association with amyloid plaques and blood vessels (Fig. 3b4–7).

### A subset of plaque-associated CD163 cells does not express classical microglial markers

As demonstrated above, the presence of Cd163 cells in the hippocampal parenchyma and associated with amyloid plaques was a pathological feature of Braak V–VI samples. Furthermore, the distribution of Cd163 cells was completely different from the classic Iba1 pattern, and the close association with blood vessels indicated that these Cd163 positive cells in AD cases could, in fact, be infiltrated monocytes. To study this possibility, we analyzed the expression of ten “specific” genes predominantly expressed in monocytes, with low expression in microglia [59]. Indeed, using recent data from single cell RNA sequencing in the human cortex [38], we confirmed that these selected genes (Additional file 1: Table S2) were enriched in “cluster 10”, corresponding to myeloid cells, versus different subsets of microglia (not shown). Thus, we determined the expression of this particular set of genes using fluidic cards. Data from these experiments were shown as heatmap (Fig. 4a1) or as gene-set-score (Fig. 4a2). As shown, the expression of these genes was highly significant (Kruskal–Wallis test, *p* = 2.932e−5; Dunn post hoc test, *p* < 0.05) increased in Braak V–VI samples, compared to Braak 0 or Braak II (Fig. 4a2), reinforcing the possible increase in myeloid infiltration. The expression of these genes was significantly correlated (Spearman, r = 0.7118 *p* < 0.0001, not shown) with the expression of genes included in the CD163-cluster defined in Fig. 1e, supporting that both groups of genes may be expressed by the same cell population.

On the other hand, the expression of classically defined microglial genes, determined in parallel, such as *TREM2, AXL, CST7* or *SALL1*, showed a small increase in Braak V–VI samples (Fig. 4b1). Interestingly, while *TREM2* expression was significantly correlated with ‘microglial’ genes, such as *SALL1* and *MEF2A* (Additional file 1: Fig. S2a, b), no correlation was observed for CD163-related genes (ie, *CD163, IfITM2, IFITM3, F13A*, or *NRA1,* Additional file 1: Fig. S2a, c), indicating that *TREM2* and *CD163* may be, preferentially, expressed by different myeloid populations. Similarly, the expression of SALL1 (specific microglial transcription factor) significantly correlated with microglial genes but not with CD163-related genes (Additional file 1: Fig. S2d).

In parallel, using the same fluidic-cards, we have also analyzed the expression of two different genes *(MRC1 and* CD169) associated with perivascular macrophages [17, 23]. As shown, no change between groups was observed (Fig. 4b2 and Additional file 1: Fig. S3c). Furthermore, Mrc1 positive cells were restricted to the perivascular space and these cells were positive for both Cd163 and Mrc1 markers (Additional file 1: Fig. S3a1, a2, b1–3), as expected from perivascular macrophages. It should be noted that the parenchymal Cd163-cells, present in Braak V–VI samples, were consistently negative for Mrc1 in all the analyzed cases (Additional file 1: Fig. S3b4–6). Therefore, it is unlikely that the parenchymal Cd163 cells, enriched in Braak V–VI samples, were of perivascular macrophage origin.

To further explore the nature of the plaque-associated Cd163 population we performed triple immunofluorescence labelling (Cd163/Iba1/Abeta or Cd163/Iba1/Trem2) in Braak V–VI hippocampal samples and combined with confocal microscopy and 3D-Imaris reconstruction (Fig. 4c, d). Our data support the existence of three different myeloid cell populations associated with Abeta plaques: (1) Iba1^+^/Trem2^+^/Cd163^**−**^ cell population (activated microglia); (2) Iba1^−^/Trem2^−^/Cd163^+^ cells (infiltrating monocytes), and (3) a subset that displayed mixed features Iba1^+^/Trem2^+^/Cd163^+^ that could represent microglial-like cells. Quantitative analysis (Fig. 4e) confirmed that Trem2^+^ (Iba1^+^) microglial cells (Cd163^−^) constituted the most abundant plaque-associated myeloid population (50–60%), 25–30% of the cells were Trem2^+^/Iba1^+^/Cd163^+^ (microglial-like cells) and, less frequently but more important, 10–15% of plaque-associated myeloid cells were Cd163^+^/Trem2^−^(Iba1^−^) which represented the infiltrated myeloid population. These data were further corroborated using double immunofluorescence labeling using Tmem119 (a pan-microglial marker) and Cd163. As shown (Additional file 1: Fig. S4a, b), three different myeloid cell populations could be distinguished: Tmem119+/Cd163− (microglial cells, 63 ± 28%), Tmem119-/Cd163+ (infiltrating monocytes, 17 ± 23%) and Tmem119+/C163+ (microglial-like cells, 19 ± 18%). It should be noted that the proportion of cells positive for Cd163 and negative for all microglial markers tested (Iba1, Trem2, and Tmem119) was similar, 10–15% from the total myeloid population.

### Infiltrated parenchymal Cd163-cells are preferentially located in the proximity of blood vessels in AD hippocampus

**Fig. 5:**
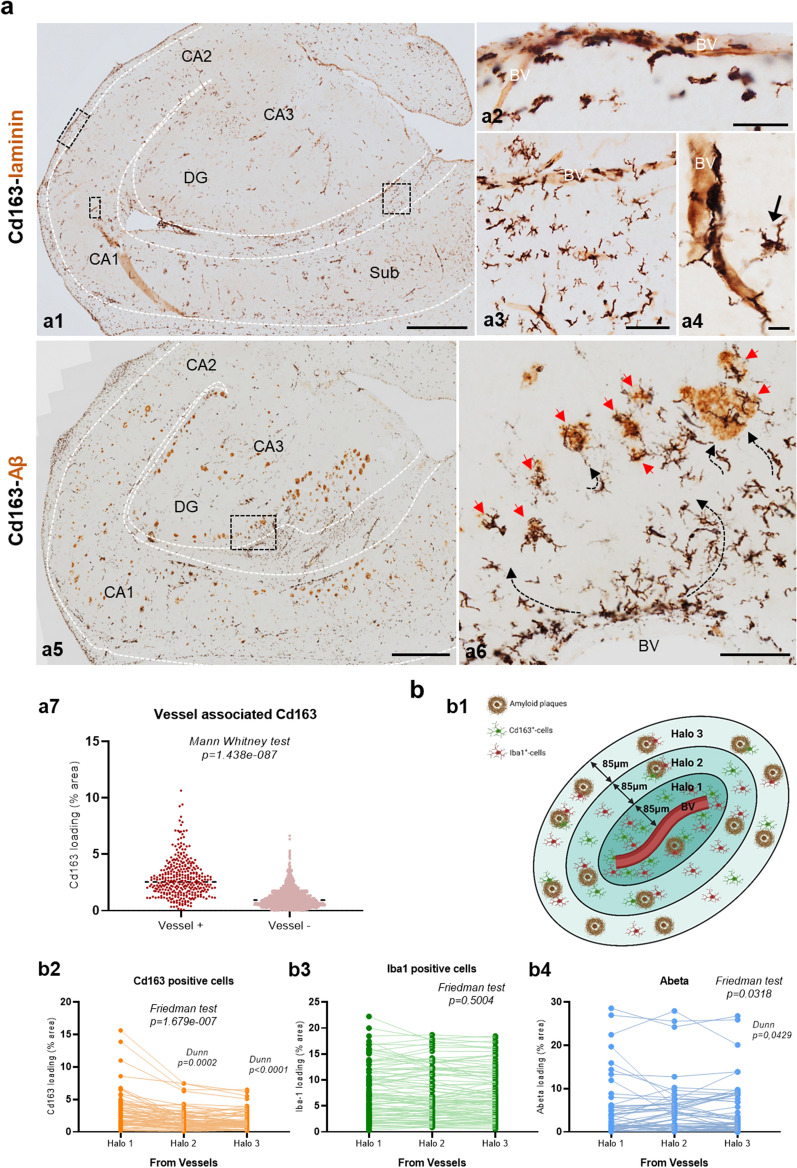
Parenchymal Cd163-positive cells are concentrated in the vicinity of blood vessels in AD hippocampus. (**a**) Double immunolabeling for Cd163 (dark brown) and, laminin or Abeta (light brown) (a1–4 and a5–6, respectively) in AD (Braak V–VI) hippocampus. Cd163-positive cells accumulated under the pia/hippocampal fissure where laminin-positive vessels were located (a1, panoramic view; boxed area, a2-a3). Higher magnification images in a3-a4 show Cd163-cells with a ramified morphology (black arrows). Double Cd163/Abeta immunostaining of the same AD hippocampus (a5, panoramic view; boxed area a6) exhibited an apparent flow direction (dashed black arrows) of Cd163-cells from blood vessels towards parenchymal Abeta plaques (red arrows). Quantitative analysis of the enriched (vessel +) and not-enriched (vessel-) laminin-positive area covered by Cd163 (percentage) is represented in a7. The results are shown individually (dots) from n = 9 Braak V–VI. Mann–Whitney U test comparison between groups. (**b**) Drawing illustrating the experimental settings (b1) to test whether microglial/myeloid cells were preferentially associated with blood vessels. As shown, the different vessels (n = 63) from AD samples (n = 9), were outlined, and three concentric circles of the same area (a fixed radius = 85 μm) were delineated surrounding each vessel. The corresponding Cd163, Iba1 and Abeta loadings were then calculated in each corresponding halo. Quantitative data indicating the loading (percentage of area) corresponding to the Cd163 (b2), Iba1 (b3) or Abeta (b4) were shown as individual matched data. The significance, shown in the figure, was tested using the Friedman test followed by the Dunn test. CA1: cornu ammonis; DG: dentate gyrus; Sub: subiculum; BV: blood vessel. Scale bars: a1 and a5, 1 mm; a3 and a6, 100 μm; a2, 50 μm; a4, 20 μm

To further support the peripheral origin of Cd163 cells in AD brains, we quantitatively evaluated the proximity of these cells to blood vessels (Fig. 5). We reasoned that if parenchymal Cd163 cells were infiltrated monocytes, they should be enriched in the proximity to blood vessels. On the contrary, if they were of microglial origin, a random distribution pattern or a preferential association with Abeta plaques should be observed. To distinguish between these possibilities, Braak V–VI hippocampal post-mortem sections were double stained for Cd163 and laminin to label blood vessels (Fig. 5a1–4) or with Abeta to label plaques (Fig. 5a5 and a6). In fact, parenchymal Cd163+ cells were not uniformly distributed in the AD hippocampus (Fig. 5a1 and a5), they concentrated under the pia and in the hippocampal fissure where blood vessels penetrate the brain from the surface. A close examination of the hippocampal fissure area revealed a preferential location of Cd163 parenchymal cells (rounded or ramified) in the white matter and in the vicinity of laminin-labeled blood vessels (see images in Figs. 5a2–4, and Additional file 1: Fig. S5). The Cd163-positive perivascular macrophages also clearly depicted the blood vessels. The Cd163/Abeta double labeling evidenced the stream of Cd163-cells apparently moving from blood vessels towards the nearby amyloid plaques (Fig. 5a6). Quantitative data (Fig. 5a7) demonstrated a highly significant association (Mann–Whitney test, *p* = 1.438e−87, n = 9 Braak V–VI cases, 337 and 1240 cells for vessel+ and −, respectively) association between Cd163 cells and blood vessels, compared to Cd163 cells not associated with laminin.

Next, we further analyze the spatial distribution of Cd163 cells related to brain blood vessels. In fact, the area positive for Cd163 was analyzed at three different distances from a particular vessel (see the diagram in Fig. 5b1; halo-1, 85 µm; halo-2, 170 µm; and halo-3, 255 µm). If Cd163 cells were infiltrating monocytes, we would expect the existence of a cell density gradient, higher in halo 1. As shown quantitatively (Fig. 5b2), there was indeed a significant gradient (Friedman test for matched data, *p* = 1.679e−007), higher in close proximity to a vessel (3.27% halo 1) and significantly decreasing with distance (2.02%, *p* = 0.0001830 Dunn test, or 1.80%, *p* = 2.709e−007 Dunn test, for halo 2 and 3, respectively, see Fig. 5b2). Using the same settings, we also quantified the distribution of Iba1 positive cells and the distribution of Abeta plaques with the distance from the blood vessels. As expected (see Fig. 5b3), Iba1 showed a homogeneous distribution in the three areas examined (Friedman test *p* = 0.5004). Furthermore, the Abeta load (Fig. 5b4) presented a small but significant (Friedman *p* = 0.03181) increase at longer distances from the vessels (4.01% halo-1 vs. 4.30% halo-3, Dunn *p* = 0.0429). In sum, our transcriptional analysis, together with immunohistological characterization, strongly supports the idea that Cd163 cells were bona fide myeloid cells that infiltrated the parenchyma, predominantly in brains with AD.

### Cd163 cell infiltration is associated with the progression and severity of AD pathology

**Fig. 6:**
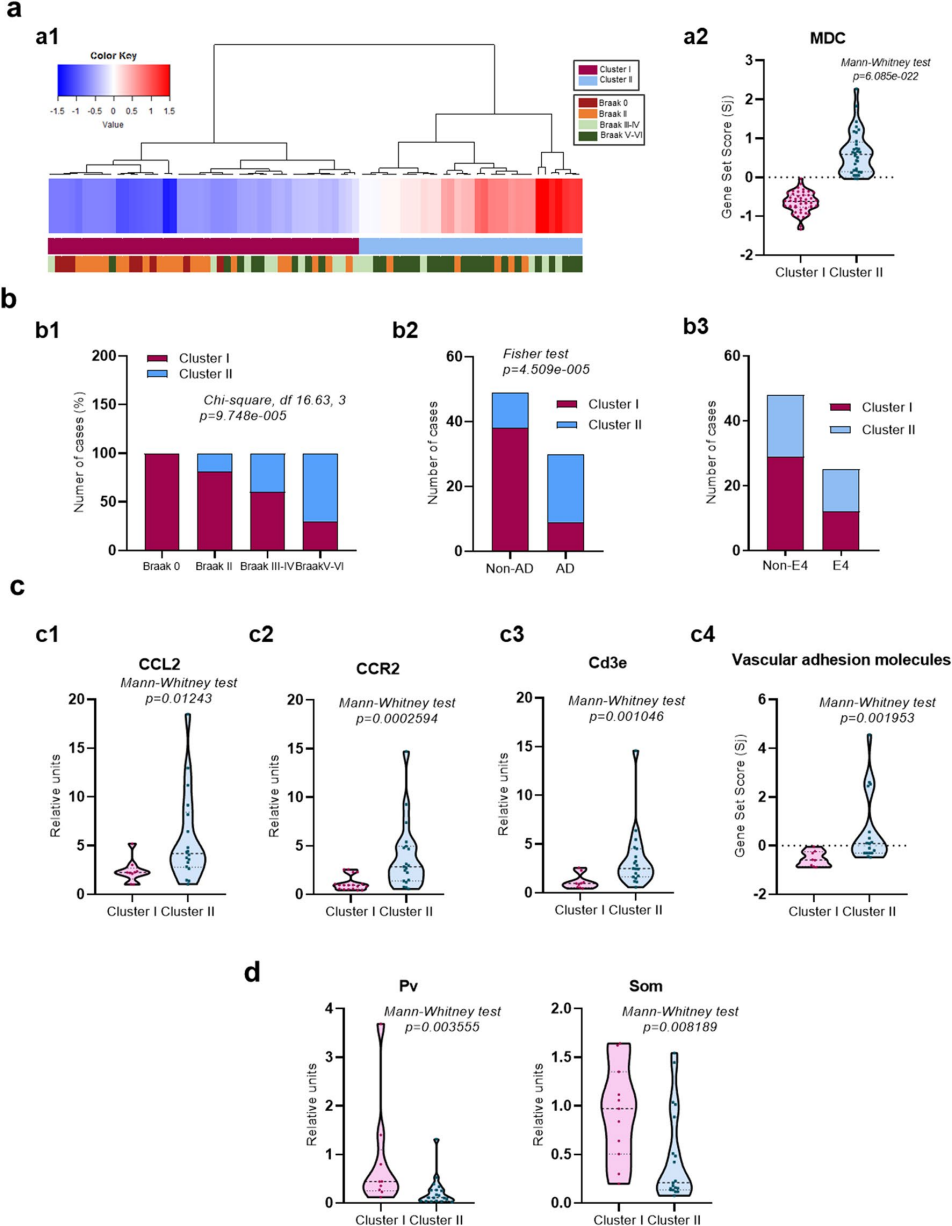
CD163-cells infiltration was associated with AD pathology and vascular endothelial activation. **(a)** Hierarchical clustering (Ward´s linkage method, Manhattan distance) analysis (a1) and gene set score (violin plots, a2) of Cd163 cluster, evaluated in the entire human cohort (n = 77 samples, comprising Braak stages 0 to VI stages). As shown, a minimal structure of two main clusters (cluster-I and cluster-II) was observed. The expression of Cd163-genes was significantly higher in cluster-II. **(b)** Using this minimal classification, we evaluated whether cluster-II was enriched in advanced Braak stages (b1), dementia (b2) or the ApoE4 genotype (b3). As shown, cluster II was enriched in BraakV–VI samples (Chi-square *p* < 0.0001) and, consequently, significantly associated (Fisher test *p* = 4.509e−005) with demented cases. No differences were observed with the ApoE4 genotype. **(c)** Since Cd163 infiltration (cluster II) was predominantly associated with Braak V–VI cases we next evaluated in this particular population the possible association with *CCL2* (c1), *CCR2* (c2) and *CD3E* (c3). As expected, we observed significant increased (Mann Whitney test; *p* < 0.05) in cluster II for all three genes tested. Furthermore, a similar analysis was performed testing the expression of vascular adhesion genes (c4). As shown, there was also a significant (Mann Whitney test, *p* < 0.05) increased levels in cluster II. **(d)** We also observed a significantly reduced expression of the GABAergic markers parvalbumin (PV) and somatostatin (SOM) in cluster II Braak V–VI individuals

As noted above, our data strongly suggested parenchymal infiltration of Cd163 cells of myeloid origin in the hippocampus of AD. However, we also noted the existence of a high degree of heterogeneity in this infiltration among different AD cases (e.g. Fig. 1e, 2c1, 4a2), indicating that not all AD brains were equally affected. Therefore, **next we investigate the possible pathological factors involved in the infiltration process. To approach this problem, we reanalyzed the expression of the Cd163-cluster in the entire sample cohort, to classify the different individuals according to the expression of the CD163-related genes. As shown (Fig. 6a), samples from all Braak stages could be classified into two main groups (Fig. 6a1). Samples with a low level of CD163-cluster expression were included in cluster I, while samples with a high expression belonged to cluster II (Fig. 6a2). As expected, a highly significant difference in expression levels was observed between both clusters (Mann–Whitney *p* = 6.085 × 10–22). Next, we tested the association between Braak stages or clinical AD and clusters I and II (Fig. 6b). We observed a significant enrichment of cluster II samples in Braak V–VI cases (Fig. 6b1, Chi-square, df 16.63, 3, *p* = 9.748e−005), however, the presence of cluster II individuals was also identified in a reduced number of Braak II or Braak III-IV samples. By classifying our sample cohort by premortem neurological evaluation in non-demented (Braak 0 to III-IV) or demented individuals (Braak V–VI Fig. 6b2), it is noteworthy that cluster II was significantly enriched in demented cases (70%) compared to non-demented individuals (30%; Fisher’s exact test *p* = 4.509e−005, OR 8.061 95% CI 2.92 to 22.42; Fig. 6b, b2). However, 30% (n = 9) of the total demented cases analyzed (n = 30) were better classified as cluster I and showed low expression. Thus, although Cd163 infiltration was clearly associated with AD, only approximately half of this population was affected.

On the other hand, we have also tested the possible association of Cd163 infiltration with other classical AD parameters (such as Apo E genotype Fig. 6b3 sex, post-mortem interval, age of death, Abeta, total accumulation of tau or phospho-Tau. Additional file 1: Figs. S6a1–a5 and S5) in the Braak V–VI population. As shown (Additional file 1: Figs. S6 and S7), except for significantly higher levels of Abeta in cluster I, we did not observe any other significant association between these parameters.

Since Cd163 infiltration was not correlated with ‘classical’ AD pathological parameters, we therefore evaluated the possible implication of different chemokines that could be released by microglia, astrocytes and other resident CNS cells to attract monocyte-derived cells [19, 59, 63]. Our results showed an evident increase in *CCL2* expression (Additional file 1: Fig. S6b, b1, b2), while *CCL3, CCL4* and *CCL5* remain constant. Furthermore, we observed that *CCL2* was significantly up-regulated in cluster II in parallel with increased expression of *CCR2* (Fig. 6 c1 and c2). Then*, CCL2* may generate an attractive response from CCR2 + cells, such as monocytes, to the parenchyma.

Furthermore, we also analyzed whether vascular endothelial activation was involved in this process. Thus, we determined the expression of the endothelial genes *SELE, ICAM2, PECAM1*, and *CDH5* and calculated the set score of the vascular adhesion molecules gene (Additional file 1: Fig. S6b3). We selected these genes because: (1) they were involved in different steps of the transmigration process (rolling, arrest, crawling, and diapedesis) [63] and (2) they were differentially expressed by endothelial cells versus other CNS resident cells [64, 65]. Interestingly, the expression of vascular adhesion molecules increased significantly in the Braak V–VI samples (Additional file 1: Fig. S6b3) and, more relevant, we also observed a clear and highly significant increase in cluster II Braak V–VI samples, compared to cluster I (Fig. 6c4). Therefore, these data suggested that monocyte-derived cells actively infiltrated the parenchyma in response to increased chemokine and endothelial activation. Similarly, we also observed an increase in the expression of the lymphocyte marker *CD3E* in cluster II (Fig. 6c3), indicating that not only monocytes but other peripheral immune cells were infiltrating in the same individuals.

Finally, we evaluated whether the possible infiltration of Cd163-positive cells was also associated with a more extensive hippocampal pathology in the Braak V–VI group. In this sense, we have recently reported the degeneration of parvalbumin (PV) and Somatostatin (SOM) positive GABAergic interneurons in the Braak V–VI samples [52]. Therefore, we also evaluated the expression of these two peptides in the same cohort. As shown (Additional file 1: Fig. S6c1-c2), we observed a significant decrease in the expression of both PV and SOM in the Braak V–VI cohort. Next, the expression of these neuronal markers was discriminated in clusters I and II and, as shown in Fig. 6d, there was a significant reduction in the expression of these two markers in those samples corresponding to cluster II. Therefore, these data strongly suggested that MDC infiltration in AD is a consequence of the severity of the pathology.

## Discussion

Whether peripheral monocytes infiltrate the brain parenchyma of AD patients and acquire a functional role is an unresolved conflictive issue. This conflict is generated by the absence of “exclusive” molecular markers that allow a clear distinction between microglia and circulating monocytes. In fact, previous reports, using AD models or human post-mortem samples, described both the presence and/or the absence of peripheral infiltration into the cerebral parenchyma [22, 33, 40]. In this work, we have reevaluated this problem by combining molecular and quantitative morphological approaches in hippocampal samples from AD patients. Our results demonstrate the presence of a small but consistent population of infiltrating monocytes in, predominantly but not exclusively, AD brains (individuals with dementia and Braak V–VI stages). The presence of infiltrating monocytes has been probed by: (i) the upregulation of genes rarely expressed by microglial cells and abundant in monocytes (such as *CD163, MSR1, CCR2, F13A1*), (ii) the increase in the proportion of positive Cd163 cells in the parenchyma from AD samples, and (iii) the clear association of Cd163 positive cells with Abeta plaques from AD samples but not with those from non-demented age-matched individuals (Braak II, CERAD B). Furthermore, and also in agreement with a previous report [40] our data show that these cells were negative for highly expressed markers in PVM, such as *MRC1*, and also ‘classical’ microglial markers, such as Iba1, Tmem119 or Trem2. More relevant, we have also shown that these Cd163 positive cells were in close proximity to blood vessels and particularly concentrated in white matter areas (such as the hippocampal fissure) whereas Iba1 cells (microglial cells) were uniformly distributed within the hippocampal parenchyma. Therefore, taken together, our data strongly support the presence of monocytes that infiltrate the AD hippocampal parenchyma. This observation is in line with those reported by [61] in the APP / PS1 models. In fact, using a fate-mapping approach to label hematopoietic monocytes, these authors demonstrated the existence of a minor population of infiltrating monocytes. However, these data are in apparent contradiction to similar fate mapping experiments of myeloid cells [47] or even in parabiosis assays [60]. Although the reasons for the discrepancies between our and these data are not clear, they could arise from the different models and/or human samples used, the different ages tested, and experimental settings. In this sense, our data also demonstrated the existence of a high degree of heterogeneity between different AD patients. In fact, not all AD cases presented clear infiltration and, in addition, the proportion of infiltrated monocytes was, at least, low on average (17% as compared to the total Iba1 population; range of 5 to 45%). In this sense, Silvin et al. [53], using snRNA-seq brain data set integration, have recently identified a relatively small similar population, disease inflammatory macrophages (DIM), presented in AD samples. As a consequence, the low proportion of infiltrating cells, together with the high variability between individuals and the possible implication of other pathologies (such as vascular comorbidities, see below) probably explain the discrepancies in the results between different models or even in AD patients.

On the other hand, *TREM2* has been classically associated with activated microglial cells in AD [20, 24] and its expression is essential for the microglial response in AD and other neurodegenerative pathologies [62]. In fact, in the presence of Abeta accumulation, mutations or total ablation of TREM2 abolish both microglial migration and activation of the DAM phenotype [24, 60]. Therefore, if the identified CD163 cluster corresponded to activated microglial cells, a close correlation between the expression of these markers should be found. However, we did not find any significant correlation between CD163-cluster genes and microglial markers (Fig. 5 and Additional file 1: Fig. S2), although *TREM2* expression showed a highly significant correlation with *SALL1* (a transcription factor expressed exclusively by microglia) [7]. Furthermore, IMARIS reconstruction of Abeta plaques or triple labeling experiments also confirm the presence of Cd163 positive and Iba1, Trem2 or Tmem119 negative cells. Thus, these data strongly support the existence of infiltrating monocytes within our AD population.

Within the different markers analyzed, CD163 (a hemoglobin scavenger receptor) constitutes the best surface receptor to identify this peripheral myeloid population. Increase in the relative abundance of Cd163 positive cells, associated with different neurodegenerative processes, such as Parkinson’s disease [40], AD (this work and [10, 40], ischemic stroke [45] and even tumor-associated macrophages (glioblastoma) [59, 64] have been extensively reported. In this sense, two different recent works [17, 36] using snRNA-seq in controls and AD samples have also identified *CD163* as a predominant altered marker in the AD cohort. In these two important works, *CD163* expression has been preferentially associated with different microglial phenotypes, such as ARM [36] or in the final AD trajectory (AD1) [17]. Therefore, these data are apparently in contradiction to those reported in this work. However, our data also demonstrated the existence of two different CD163 populations, negative (10–18%) and positive for Iba1,Trem2 and/or Tmem119 (20–30% see Fig. 4 and Additional file 1: Fig. S4). Therefore, it is possible that this Cd163 and Iba1, Trem2 or Tmem119 positive population corresponds to ARM/AD1 cells, while the Cd163 positive /Iba1,Trem2 or Tmem119 negative cells were infiltrating monocytes. On the other hand, we cannot exclude that both populations were indeed infiltrated monocytes at different differentiation stages. In this sense, it has been proposed that the CNS environment could reprogram infiltrating monocytes towards a microglial phenotype [2] expressing most, but not all, “classical” microglial markers.

On the other hand, the present results do not allow us to conclude whether monocyte infiltration is beneficial or detrimental to AD pathology. In fact, infiltrating monocytes have been postulated to show high phagocytic capacity [56]. Furthermore, Ccr2 ablation increases the Abeta pathology [25] and it has been demonstrated that enhanced recruitment of cerebral Abeta-associated monocytes diminished neuropathology and preserved cognitive function in amyloidogenic models [28, 29]. Similarly, blocking PD-L1 signaling in AD models increased monocyte-derived cell infiltration into the cerebral parenchyma, reducing brain pathology [1, 48]. As a consequence, monocyte infiltration within the cerebral parenchyma might, to some extent, alleviate the pathology of AD. In this sense, our data also demonstrate the close association between Cd163 cells and Abeta plaques in AD samples. Furthermore, several markers associated with this population can participate in a phagocytic response, such as *CD163, CD68* and *MSR1* [4, 34, 55]. However, our data also showed that, in our AD population, the presence of infiltrating Cd163 positive cells is also correlated with lower levels of specific GABAergic interneuronal makers, such as PV and SOM. Based on our own data [46, 52], the decrease in the expression of GABAergic markers constitutes a “proxi” reflecting the neurodegenerative process observed in both AD cases and Abeta models. Consequently, a decrease in the expression of PV and SOM should reflect a reduction in GABAergic interneurons and, consequently, reflects a major neurological pathology. Therefore, although infiltrating monocytes could indeed have higher Abeta phagocytic capacity or a beneficial immune response, in our AD cohort, infiltration seems to be a consequence of a higher AD pathology (see below). In fact, we did not observe significant differences in tau accumulation or tau phosphorylation between AD samples with high or low Cd163 infiltration.

In consonance with these data, it has been demonstrated the increase in the soluble form of Cd163 in cerebrospinal fluid (CSF) in late Parkinson’s disease (PD) cases that was associated with cognitive decline condition [37]. In fact, high levels of soluble Cd163 in CSF indicated a bad prognosis of PD [27]. Therefore, the monocyte infiltration seems to be preferentially associated with advanced stages of the neurodegenerative diseases. However, these data do not exclude the proposed beneficial role of monocyte infiltration in AD pathology, as previously reported [28, 29]. In fact, enhancing brain infiltrating monocytes (with high Abeta-removing capacity) in earlier stages of the pathology could indeed be a therapeutic approach to reduce Abeta accumulation in brains with AD.

Finally, our data also indicate that monocyte infiltration is not the cause but a consequence of AD pathology. In fact, approximately 30% of the AD cases analyzed with high Abeta and Tau pathology had low or no myeloid infiltration. Furthermore, we also identified non-demented Braak II or Braak III–IV cases with high infiltration levels. Therefore, it is possible that other comorbidities, associated with aging or other pathologies (i.e. vascular pathology), could be responsible for the observed infiltration. In this sense, our data clearly suggest a clear relationship with endothelial activation and possible damage to the BBB. In fact, we observed an increase in the expression of intercellular adhesion genes in the Braak V–VI population, and, more importantly, the expression of these vascular adhesion genes increased in samples with high levels of CD163 expression. Thus, vascular endothelial activation, associated with AD or other pathologies, may be responsible for the recruitment and infiltration of inflammatory cells into the cerebral parenchyma. These data are consistent with the diminished integrity of the BBB and endothelial activation associated with cerebral amyloid angiopathy, vascular dementia, and AD [14, 39, 54] Furthermore, we have also recently reported the existence of microglial degeneration in specific areas (dentate gyrus) of approximately 50% of AD samples [51]. Since microglial depopulation also induces monocyte infiltration [9], microglial degeneration could also be a pathological factor that induces higher monocyte infiltration. Therefore, our data strongly suggest that monocyte infiltration is a consequence, not the cause, of AD and/or AD-associated pathology.

In summary, our data strongly support the existence of a small proportion (over total microglial cells) of monocytes that infiltrate the AD brain parenchyma. The recruitment of monocytes could be a consequence rather than the cause of the severity of the disease. Whether invading monocyte-derived cells are detrimental or beneficial to disease progression remains elusive. Unrevealing this critical issue will open new opportunities for AD therapeutics.

## Supplementary Information

**Additional file 1:** Additional Figures and Tables.

## Data Availability

All study data have been provided in paper and supplementary materials.

## References

[1] Baruch K, Deczkowska A, Rosenzweig N, Tsitsou-Kampeli A, Sharif AM, Matcovitch-Natan O, Kertser A, David E, Amit I, Schwartz M (2016). PD-1 immune checkpoint blockade reduces pathology and improves memory in mouse models of Alzheimer’s disease. Nat Med.

[2] Bennett ML, Bennett FC (2020). The influence of environment and origin on brain resident macrophages and implications for therapy. Nat Neurosci.

[3] Bogie JFJ, Stinissen P, Hendriks JJA (2014). Macrophage subsets and microglia in multiple sclerosis. Acta Neuropathol.

[4] Bonilla DL, Bhattacharya A, Sha Y, Xu Y, Xiang Q, Kan A, Jagannath C, Komatsu M, Eissa NT (2013). Autophagy regulates phagocytosis by modulating the expression of scavenger receptors. Immunity.

[5] Böttcher C, Schlickeiser S, Sneeboer MAM, Kunkel D, Knop A, Paza E, Fidzinski P, Kraus L, Snijders GJL, Kahn RS, Schulz AR, Mei HE, Hol EM, Siegmund B, Glauben R, Spruth EJ, de Witte LD, Priller J (2019). Human microglia regional heterogeneity and phenotypes determined by multiplexed single-cell mass cytometry. Nat Neurosci.

[6] Brezovakova V, Valachova B, Hanes J, Novak M, Jadhav S (2018). Dendritic cells as an alternate approach for treatment of neurodegenerative disorders. Cell Mol Neurobiol.

[7] Buttgereit A, Lelios I, Yu X, Vrohlings M, Krakoski NR, Gautier EL, Nishinakamura R, Becher B, Greter M (2016). Sall1 is a transcriptional regulator defining microglia identity and function. Nat Immunol.

[8] Chen X, Holtzman DM (2022). Emerging roles of innate and adaptive immunity in Alzheimer’s disease. Immunity.

[9] Cronk JC, Filiano AJ, Louveau A, Marin I, Marsh R, Ji E, Goldman DH, Smirnov I, Geraci N, Acton S, Overall CC, Kipnis J (2018). Peripherally derived macrophages can engraft the brain independent of irradiation and maintain an identity distinct from microglia. J Exp Med.

[10] Dal Bianco A, Bradl M, Frischer J, Kutzelnigg A, Jellinger K, Lassmann H (2008). Multiple sclerosis and Alzheimer’s disease. Ann Neurol.

[11] Fernandez-Valenzuela JJ, Sanchez-Varo R, Muñoz-Castro C, de Castro V, Sanchez-Mejias E, Navarro V, Jimenez S, Nuñez-Diaz C, Gomez-Arboledas A, Moreno-Gonzalez I, Vizuete M, Davila JC, Vitorica J, Gutierrez A (2020). Enhancing microtubule stabilization rescues cognitive deficits and ameliorates pathological phenotype in an amyloidogenic Alzheimer’s disease model. Sci Rep.

[12] Fernández Zapata C, Giacomello G, Spruth EJ, Middeldorp J, Gallaccio G, Dehlinger A, Dames C, Leman JKH, van Dijk RE, Meisel A, Schlickeiser S, Kunkel D, Hol EM, Paul F, Parr MK, Priller J, Böttcher C (2022). Differential compartmentalization of myeloid cell phenotypes and responses towards the CNS in Alzheimer’s disease. Nat Commun.

[13] Ferretti MT, Merlini M, Späni C, Gericke C, Schweizer N, Enzmann G, Engelhardt B, Kulic L, Suter T, Nitsch RM (2016). T-cell brain infiltration and immature antigen-presenting cells in transgenic models of Alzheimer’s disease-like cerebral amyloidosis. Brain Behav Immun.

[14] Fisher RA, Miners JS, Love S (2022). Pathological changes within the cerebral vasculature in Alzheimer’s disease: New perspectives. Brain Pathol.

[15] Friedman BA, Srinivasan K, Ayalon G, Meilandt WJ, Lin H, Huntley MA, Cao Y, Lee SH, Haddick PCG, Ngu H, Modrusan Z, Larson JL, Kaminker JS, van der Brug MP, Hansen D, v. (2018). Diverse brain myeloid expression profiles reveal distinct microglial activation states and aspects of Alzheimer’s disease not evident in mouse models. Cell Rep.

[16] GAREY LJ, (1997). Atlas of the human brain. J Anat.

[17] Gerrits E, Brouwer N, Kooistra SM, Woodbury ME, Vermeiren Y, Lambourne M, Mulder J, Kummer M, Möller T, Biber K, den Dunnen WFA, de Deyn PP, Eggen BJL, Boddeke EWGM (2021). Distinct amyloid-β and tau-associated microglia profiles in Alzheimer’s disease. Acta Neuropathol.

[18] Greenhalgh AD, David S, Bennett FC (2020). Immune cell regulation of glia during CNS injury and disease. Nat Rev Neurosci.

[19] Guedes JR, Lao T, Cardoso AL, El Khoury J (2018). Roles of microglial and monocyte chemokines and their receptors in regulating Alzheimer’s disease-associated amyloid-β and Tau pathologies. Front Neurol.

[20] Hansen DV, Hanson JE, Sheng M (2018). Microglia in Alzheimer’s disease. J Cell Biol.

[21] Hohsfield LA, Najafi AR, Ghorbanian Y, Soni N, Hingco EE, Kim SJ, Jue AD, Swarup V, Inlay MA, Green KN (2020). Effects of long-term and brain-wide colonization of peripheral bone marrow-derived myeloid cells in the CNS. J Neuroinflammation.

[22] Jin F, Xi Y, Xie D, Wang Q (2022). Comprehensive analysis reveals a 5-gene signature and immune cell infiltration in Alzheimer’s disease with qPCR validation. Front Genet.

[23] Jordão MJC, Sankowski R, Brendecke SM, Sagar LG, Tai YH, Tay TL, Schramm E, Armbruster S, Hagemeyer N, Groß O, Mai D, Çiçek Ö, Falk T, Kerschensteiner M, Grün D, Prinz M (2019). Single-cell profiling identifies myeloid cell subsets with distinct fates during neuroinflammation. Science.

[24] Keren-Shaul H, Spinrad A, Weiner A, Matcovitch-Natan O, Dvir-Szternfeld R, Ulland TK, David E, Baruch K, Lara-Astaiso D, Toth B, Itzkovitz S, Colonna M, Schwartz M, Amit I (2017). A unique microglia type associated with restricting development of Alzheimer’s disease. Cell.

[25] el Khoury J, Toft M, Hickman SE, Means TK, Terada K, Geula C, Luster AD (2007). Ccr2 deficiency impairs microglial accumulation and accelerates progression of Alzheimer-like disease. Nat Med.

[26] Kierdorf K, Masuda T, Jordão MJC, Prinz M (2019). Macrophages at CNS interfaces: ontogeny and function in health and disease. Nat Rev Neurosci.

[27] Konstantin Nissen S, Farmen K, Carstensen M, Schulte C, Goldeck D, Brockmann K, Romero-Ramos M (2022). Changes in CD163+, CD11b+, and CCR2+ peripheral monocytes relate to Parkinson’s disease and cognition. Brain Behav Immun.

[28] Koronyo Y, Salumbides BC, Sheyn J, Pelissier L, Li S, Ljubimov V, Moyseyev M, Daley D, Fuchs DT, Pham M, Black KL, Rentsendorj A, Koronyo-Hamaoui M (2015) Therapeutic effects of glatiramer acetate and grafted CD115^+^ monocytes in a mouse model of Alzheimer’s disease. Brain 138:2399–2422. 10.1093/BRAIN/AWV150 10.1093/brain/awv150 PMC4840949 26049087

[29] Koronyo-Hamaoui M, Sheyn J, Hayden EY, Li S, Fuchs DT, Regis GC, Lopes DHJ, Black KL, Bernstein KE, Teplow DB, Fuchs S, Koronyo Y, Rentsendorj A (2020) Peripherally derived angiotensin converting enzyme-enhanced macrophages alleviate Alzheimer-related disease. Brain 143:336–358. 10.1093/BRAIN/AWZ364 10.1093/brain/awz364 PMC6935752 31794021

[30] Livak KJ, Schmittgen TD (2001). Analysis of relative gene expression data using real-time quantitative PCR and the 2(-Delta Delta C(T)) Method. Methods.

[31] Lund H, Pieber M, Parsa R, Han J, Grommisch D, Ewing E, Kular L, Needhamsen M, Espinosa A, Nilsson E, Överby AK, Butovsky O, Jagodic M, Zhang XM, Harris RA (2018). Competitive repopulation of an empty microglial niche yields functionally distinct subsets of microglia-like cells. Nat Commun.

[32] Merlini M, Kirabali T, Kulic L, Nitsch RM, Ferretti MT (2018). Extravascular CD3+ T cells in brains of Alzheimer disease patients correlate with tau but not with amyloid pathology: An immunohistochemical study. Neurodegener Dis.

[33] Monoranu CM, Hartmann T, Strobel S, Heinsen H, Riederer P, DIstel L, Bohnert S (2021). Is there any evidence of monocytes involvement in Alzheimer’s disease? a pilot study on human postmortem brain. J Alzheimers Dis Rep.

[34] Murray PJ, Wynn TA (2011). Protective and pathogenic functions of macrophage subsets. Nat Rev Immunol.

[35] Navarro V, Sanchez-Mejias E, Jimenez S, Muñoz-Castro C, Sanchez-Varo R, Davila JC, Vizuete M, Gutierrez A, Vitorica J (2018). Microglia in Alzheimer’s disease: activated, dysfunctional or degenerative. Front Aging Neurosci.

[36] Nguyen AT, Wang K, Hu G, Wang X, Miao Z, Azevedo JA, Suh ER, van Deerlin VM, Choi D, Roeder K, Li M, Lee EB (2020). APOE and TREM2 regulate amyloid-responsive microglia in Alzheimer’s disease. Acta Neuropathol.

[37] Nissen SK, Ferreira SA, Nielsen MC, Schulte C, Shrivastava K, Hennig D, Etzerodt A, Graversen JH, Berg D, Maetzler W, Panhelainen A, Møller HJ, Brockmann K, Romero-Ramos M (2021). Soluble CD163 changes indicate monocyte association with cognitive deficits in Parkinson’s disease. Movement Disord.

[38] Olah M, Menon V, Habib N, Taga MF, Ma Y, Yung CJ, Cimpean M, Khairallah A, Coronas-Samano G, Sankowski R, Grün D, Kroshilina AA, Dionne D, Sarkis RA, Cosgrove GR, Helgager J, Golden JA, Pennell PB, Prinz M, Vonsattel JPG, Teich AF, Schneider JA, Bennett DA, Regev A, Elyaman W, Bradshaw EM, de Jager PL (2020). Single cell RNA sequencing of human microglia uncovers a subset associated with Alzheimer’s disease. Nat Commun.

[39] Parodi-Rullán RM, Javadov S, Fossati S (2021). Dissecting the crosstalk between endothelial mitochondrial damage, vascular inflammation, and neurodegeneration in cerebral amyloid angiopathy and Alzheimer’s disease. Cells.

[40] Pey P, Pearce RKB, Kalaitzakis ME, Griffin WST, Gentleman SM (2014). Phenotypic profile of alternative activation marker CD163 is different in Alzheimer’s and Parkinson’s disease. Acta Neuropathol Commun.

[41] Plemel JR, Stratton JA, Michaels NJ, Rawji KS, Zhang E, Sinha S, Baaklini CS, Dong Y, Ho M, Thorburn K, Friedman TN, Jawad S, Silva C, Caprariello A, v., Hoghooghi V, Yue J, Jaffer A, Lee K, Kerr BJ, Midha R, Stys PK, Biernaskie J, Wee Yong V, (2020). Microglia response following acute demyelination is heterogeneous and limits infiltrating macrophage dispersion. Sci Adv.

[42] Prinz M, Priller J (2017). The role of peripheral immune cells in the CNS in steady state and disease. Nat Neurosci.

[43] Prinz M, Jung S, Priller J (2019). Microglia biology: one century of evolving concepts. Cell.

[44] Prokop S, Miller KR, Drost N, Handrick S, Mathur V, Luo J, Wegner A, Wyss-Coray T, Heppner FL (2015). Impact of peripheral myeloid cells on amyloid-β pathology in Alzheimer’s disease-like mice. J Exp Med.

[45] Rajan WD, Wojtas B, Gielniewski B, Miró-Mur F, Pedragosa J, Zawadzka M, Pilanc P, Planas AM, Kaminska B (2020). Defining molecular identity and fates of CNS-border associated macrophages after ischemic stroke in rodents and humans. Neurobiol Dis.

[46] Ramos B, Baglietto-Vargas D, del Rio JC, Moreno-Gonzalez I, Santa-Maria C, Jimenez S, Caballero C, Lopez-Tellez JF, Khan ZU, Ruano D, Gutierrez A, Vitorica J (2006). Early neuropathology of somatostatin/NPY GABAergic cells in the hippocampus of a PS1xAPP transgenic model of Alzheimer’s disease. Neurobiol Aging.

[47] Reed-Geaghan EG, Croxford AL, Becher AL, Landreth GE (2020). Plaque-associated myeloid cells derive from resident microglia in an Alzheimer’s disease model. J Exp Med.

[48] Rosenzweig N, Dvir-Szternfeld R, Tsitsou-Kampeli A, Keren-Shaul H, Ben-Yehuda H, Weill-Raynal P, Cahalon L, Kertser A, Baruch K, Amit I, Weiner A, Schwartz M (2019). PD-1/PD-L1 checkpoint blockade harnesses monocyte-derived macrophages to combat cognitive impairment in a tauopathy mouse model. Nat Commun.

[49] Rossi F, Lewis C (2018). Microglia’s heretical self-renewal. Nat Neurosci.

[50] Rustenhoven J, Smith AM, Smyth LC, Jansson D, Scotter EL, Swanson MEV, Aalderink M, Coppieters N, Narayan P, Handley R, Overall C, Park TIH, Schweder P, Heppner P, Curtis MA, Faull RLM, Dragunow M (2018). PU.1 regulates Alzheimer’s disease-associated genes in primary human microglia. Mol Neurodegener.

[51] Sanchez-Mejias E, Navarro V, Jimenez S, Sanchez-Mico M, Sanchez-Varo R, Nuñez-Diaz C, Trujillo-Estrada L, Davila JC, Vizuete M, Gutierrez A, Vitorica J (2016). Soluble phospho-tau from Alzheimer’s disease hippocampus drives microglial degeneration. Acta Neuropathol.

[52] Sanchez-Mejias E, Nuñez-Diaz C, Sanchez-Varo R, Gomez-Arboledas A, Garcia-Leon JA, Fernandez-Valenzuela JJ, Mejias-Ortega M, Trujillo-Estrada L, Baglietto-Vargas D, Moreno-Gonzalez I, Davila JC, Vitorica J, Gutierrez A (2020). Distinct disease-sensitive GABAergic neurons in the perirhinal cortex of Alzheimer’s mice and patients. Brain Pathol.

[53] Silvin A, Uderhardt S, Piot C, da Mesquita S, Yang K, Geirsdottir L (2022). Dual ontogeny of disease-associated microglia and disease inflammatory macrophages in aging and neurodegeneration. Immunity.

[54] Situ M, Citalan-Madrid AF, Stamatovic SM, Keep RF, Andjelkovic AV (2022). Transcriptomic profile of blood-brain barrier remodeling in cerebral amyloid angiopathy. Front Cell Neurosci.

[55] Swanson MEV, Murray HC, Ryan B, Faull RLM, Dragunow M, Curtis MA (2020). Quantitative immunohistochemical analysis of myeloid cell marker expression in human cortex captures microglia heterogeneity with anatomical context. Sci Rep.

[56] Thériault P, Elali A, Rivest S (2015). The dynamics of monocytes and microglia in Alzheimer’s disease. Alzheimers Res Ther.

[57] Unger MS, Marschallinger J, Kaindl J, Klein B, Johnson M, Khundakar AA, Roßner S, Heneka MT, Couillard-Despres S, Rockenstein E, Masliah E, Attems J, Aigner L (2018). Doublecortin expression in CD8+ T-cells and microglia at sites of amyloid-β plaques: A potential role in shaping plaque pathology?. Alzheimers Dement.

[58] Varvel NH, Grathwohl SA, Degenhardt K, Resch C, Bosch A, Jucker M, Neher JJ (2015). Replacement of brain-resident myeloid cells does not alter cerebral amyloid-β deposition in mouse models of Alzheimer’s disease. J Exp Med.

[59] Venteicher AS, Tirosh I, Hebert C, Yizhak K, Neftel C, Filbin MG, Hovestadt V, Escalante LE, Shaw ML, Rodman C, Gillespie SM, Dionne D, Luo CC, Ravichandran H, Mylvaganam R, Mount C, Onozato ML, Nahed BV, Wakimoto H, Curry WT, Iafrate AJ, Rivera MN, Frosch MP, Golub TR, Brastianos PK, Getz G, Patel AP, Monje M, Cahill DP, Rozenblatt-Rosen O, Louis DN, Bernstein BE, Regev A, Suvà ML (2017). Decoupling genetics, lineages, and microenvironment in IDH-mutant gliomas by single-cell RNA-seq. Science.

[60] Wang Y, Ulland TK, Ulrich JD, Song W, Tzaferis JA, Hole JT, Yuan P, Mahan TE, Shi Y, Gilfillan S, Cella M, Grutzendler J, DeMattos RB, Cirrito JR, Holtzman DM, Colonna M (2016). TREM2-mediated early microglial response limits diffusion and toxicity of amyloid plaques. J Exp Med.

[61] Yan P, Kim KW, Xiao Q, Ma X, Czerniewski LR, Rawnsley DR, Yan Y, Randolph GJ, Epelman S, Lee JM, Diwan A (2022). Peripheral monocyte-derived cells counter amyloid plaque pathogenesis in a mouse model of Alzheimer’s disease. J Clin Invest.

[62] Yeh FL, Hansen DV, Sheng M (2017). TREM2, microglia, and neurodegenerative diseases. Trends Mol Med.

[63] Zenaro E, Piacentino G, Constantin G (2017). The blood-brain barrier in Alzheimer’s disease. Neurobiol Dis.

[64] Zhang H, Zhang N, Wu W, Wang Z, Dai Z, Liang X, Zhang L, Peng Y, Luo P, Zhang J, Liu Z, Cheng Q, Liu Z (2022). Pericyte mediates the infiltration, migration, and polarization of macrophages by CD163/MCAM axis in glioblastoma. iScience.

[65] Zhang Y, Sloan SA, Clarke LE, Caneda C, Plaza CA, Blumenthal PD, Vogel H, Steinberg GK, Edwards MSB, Li G, Duncan JA, Cheshier SH, Shuer LM, Chang EF, Grant GA, Gephart MGH, Barres BA (2016). Purification and characterization of progenitor and mature human astrocytes reveals transcriptional and functional differences with mouse. Neuron.

[66] Zondler L, Müller K, Khalaji S, Bliederhäuser C, Ruf WP, Grozdanov V, Thiemann M, Fundel-Clemes K, Freischmidt A, Holzmann K, Strobel B, Weydt P, Witting A, Thal DR, Helferich AM, Hengerer B, Gottschalk KE, Hill O, Kluge M, Ludolph AC, Danzer KM, Weishaupt JH (2016) Peripheral monocytes are functionally altered and invade the CNS in ALS patients. Acta Neuropathol 132:391–411. 10.1007/S00401-016-1548-Y 10.1007/s00401-016-1548-y 26910103

