# Supplementary material for: Monocyte-derived cells invade brain parenchyma and amyloid plaques in human Alzheimer’s disease hippocampus
Source: Acta Neuropathol Commun. 2023 Feb 28;11:31. doi: 10.1186/s40478-023-01530-z (PMC9976401; doi:10.1186/s40478-023-01530-z)
Supplement: Supplementary file 1 — Additional file 1: Additional Figures and Tables. [file 40478_2023_1530_MOESM1_ESM.pdf]

Additional File for:

Monocyte-derived cells invade brain parenchyma and amyloid plaques in human Alzheimer's disease hippocampus

Clara Muñoz-Castro[1,2,4*], Marina Mejias-Ortega[3,4*], Elisabeth Sanchez-Mejias[3,4], Victoria Navarro[1,2,4], Laura Trujillo-Estrada[3,4], Sebastian Jimenez[1,2,4], Juan Antonio Garcia-Leon[3,4], Juan Jose Fernandez-Valenzuela[3,4], Maria Virtudes Sanchez-Mico[1,2,4], Carmen Romero-Molina[1,2,4], Ines Moreno-Gonzalez[3,4], David Baglietto-Vargas[3,4], Marisa Vizuete[1,2,4], Antonia Gutierrez[3,4‡] and Javier Vitorica[1,2,4‡]

1-Dpto. Bioquimica y Biologia Molecular, Facultad de Farmacia, Universidad de Sevilla, 41012 Sevilla, Spain.
2-Instituto de Biomedicina de Sevilla (IBiS)-Hospital Universitario Virgen del Rocío/CSIC/Universidad de Sevilla, 41013 Sevilla, Spain.
3-Dpto. Biologia Celular, Genetica y Fisiologia, Instituto de Investigación Biomedica de Malaga-IBIMA, Facultad de Ciencias, Universidad de Malaga, 29071 Malaga, Spain
4-Centro de Investigacion Biomedica en Red sobre Enfermedades Neurodegenerativas (CIBERNED), 28031 Madrid, Spain
* Contributed equally as co-first authors
‡ Co-seniors corresponding authors

Corresponding authors:

Javier Vitorica
Dpto. Bioquimica y Biologia Molecular
Facultad de Farmacia. Universidad de Sevilla
C/ Prof. Garcia Gonzalez 2, Sevilla 41012, Spain
Telephone: 34-95-4556770
E-mail: vitorica@us.es

Antonia Gutierrez
Dpto. Biologia Celular, Genetica y Fisiologia
Facultad de Ciencias, Universidad de Malaga
Campus de Teatinos s/n, Malaga 29071, Spain
Telephone: 34-95-2133344
E-mail: agutierrez@uma.es

ADDITIONAL FIGURES

Figure S1. Expression analysis of myeloid/microglial markers in post-mortem human samples with different AD pathology. **(a)** Braak stage-dependent variations on myeloid gene expression tested (by qPCR) in 66 different human samples (Braak 0, n=8; Braak II, n=21; Braak III-IV, n=9 and Braak V-VI, n=28). Statistical differences (indicated in the Figure) between Braak groups were analyzed using Kruskall-Kallis followed by Dunn post hoc test. **(b)** Hierarchical clustering analysis (Ward´s linkage method, Manhattan distance) of genes with significantly altered expression.

Figure S2. *TREM2 and SALL1* expression correlate exclusively with microglial-specific genes in Braak VI samples. Spearman's correlation between *TREM2* expression and microglial or CD163-related genes was resumed in **(a)** or individually, as dot plots, shown in panels **b** and **c**. Whereas *TREM2* expression was significantly correlated with microglial genes (*SALL1, MEF2A*), there was no correlation with any of the CD163-related genes tested. *SALL1* expression correlated with microglial genes (*AXL, CST7* and *MEF2A*) and there was no correlation with CD163-related genes **(d)** . The expression of *TREM2, SALL1* and different microglial and *CD163* specific genes was assayed in parallel, using fluidic cards.

Figure S3. Perivascular macrophages are restricted to blood vessels. **(a)** Immunostaining for Mannose Receptor 1 (Mrc1) in age-matched controls (Braak II samples). Mrc1-positive cells were limited to blood vessels (a1, boxed area a2). Higher magnification images in a2-3 show non-ramified Mrc1 cells in close association with blood vessels. **(b)** Confocal analysis of cells double labelled with Mrc1 (b1) and Cd163 (b2) in Braak VI samples. As shown, vascular macrophages were clearly positive for both markers (b3), while infiltrating Cd163 positive cells (b5) were negative for Mrc1 (b4 and b6). **(c)** Analysis of the expression of the PVM markers MRC1 and CD169 genes. As expected, no significant Braak-stage-dependent variations were observed, corroborating the absence of PVM infiltration. *Scale bars: a1: 200 µm; a2, 100 µm; a3, 10 µm; b1-b6, 10 µm.*

Figure S4. Myeloid cells heterogeneity in AD hippocampus. **(a)** Confocal microscopy of triple Tmem119/Cd163/Abeta immunofluorescence in AD hippocampus reveals different subsets of plaque-associated myeloid cells, including Tmem119+ microglia (a1, red arrows in a3), Cd163+ infiltrated cells (a2, green arrow in a3) and CD63+/Tmem119+ microglial-like cells (white arrow in a3). **(b)** Graph shows the cell counting of the different microglia/myeloid cells per frame from n=6 Braak V-VI cases (100-150 cells/case). As shown, Tmem119+/Cd163- microglia represented the largest cell subset in comparison with Cd163+ infiltrating cells or Tmem119+/Cd163+ microglial-like cells. *Scale bars: a1 and a2, 20 µm; a3, 10 µm.*

Figure S5. Cd163+ cells are abundant in the vicinity of blood vessels in white matter areas in AD hippocampus. Representative images of Braak II CERAD B (a1-3; control) and Braak V-VI CERAD C (b1-3, c1-4; AD) hippocampus immunostained for Cd163 (dark brown) and for Abeta deposits (OC antibody, light brown). Cd163+ cells of controls cases were restricted to blood vessels wall (perivascular cells) in the white matter areas (a1, panoramic view; boxed area a2-a3, grey arrows). Double Cd163/Abeta immunostaining of two different AD individuals (b1 and c1, hippocampal panoramic views; boxed area b2-3 and c2) exhibited also numerous infiltrated Cd163+ cells with ameboid morphology close located to blood vessels in the white matter (c3, red arrows). No Abeta deposits were detected in the white matter (higher magnification images in b2-3, c3-4). bv: blood vessel, hf: hippocampal fissure, agb: angular bundle, DG: dentate gyrus, CA: cornus Ammonis, Sub: subiculum. *Scale bar: a1, b1 and c1, 2 mm; a2-3 and b2-3, 200 µm; c2, 500 µm; c3-4, 100 µm.*

Figure S6. (a) Distribution between Cd163 cluster I and II of Braak V-VI cases according to sex (a1), post-mortem delay (a2), age of exitus (a3), tau pathology (a4, data from Supplemental. Fig. 5) or total Abeta monomers (a5, data from Supplemental. Fig 5)**.** As shown, individuals in cluster I and II were similar for all parameters tested except for a significant decrease in total Abeta content. The relevance of this difference is currently not understood. **(b)** Expression analysis of *CCL2,3,4* and *5* chemokines (b1) in post-mortem human Braak II and Braak V-VI samples. As shown, only the expression of *CCL2* was significantly

increased (Mann Whitney test; $p<0.05$) in Braak V-VI cohort. This increase was further corroborated testing *CCL2* expression through Braak 0 to Braak V-VI samples (b2). b3) Braak dependent variations of the gene set score of vascular adhesion genes (*SELE, ICAM2, PECAM1,* and *CDH5*). Note the clear up-regulation of these genes mainly in Braak V-VI subjects. **(c)** Braak stage-dependent variations on the expression of the GABAergic markers parvalbumin (PV, c1) or somatostatin (SOM, c2) (Braak 0, n=8; Braak II, n=21; Braak III-IV, n=9 and Brrak V-VI, n=28). Statistical differences (indicated in the Figure) between Braak groups were analyzed using Kruskall-Kallis followed by Dunn post hoc test.

Figure S7. Representative uncropped western blots of Abeta, Tau and phospho-Tau. **(a)** Total Abeta, extracted with SDS plus Urea, was determined using mAb 82E1. The monomeric Abeta (4.5kDa band) was quantitatively determined. **(b-d)** Total Tau and AT8 or AT100 positive phospho-Tau was also quantitatively determined. As shown, Tau was heavily aggregated in, predominantly, Braak V-VI samples. Thus, the whole lane (from 50kDa to the origin) for each sample was quantified.

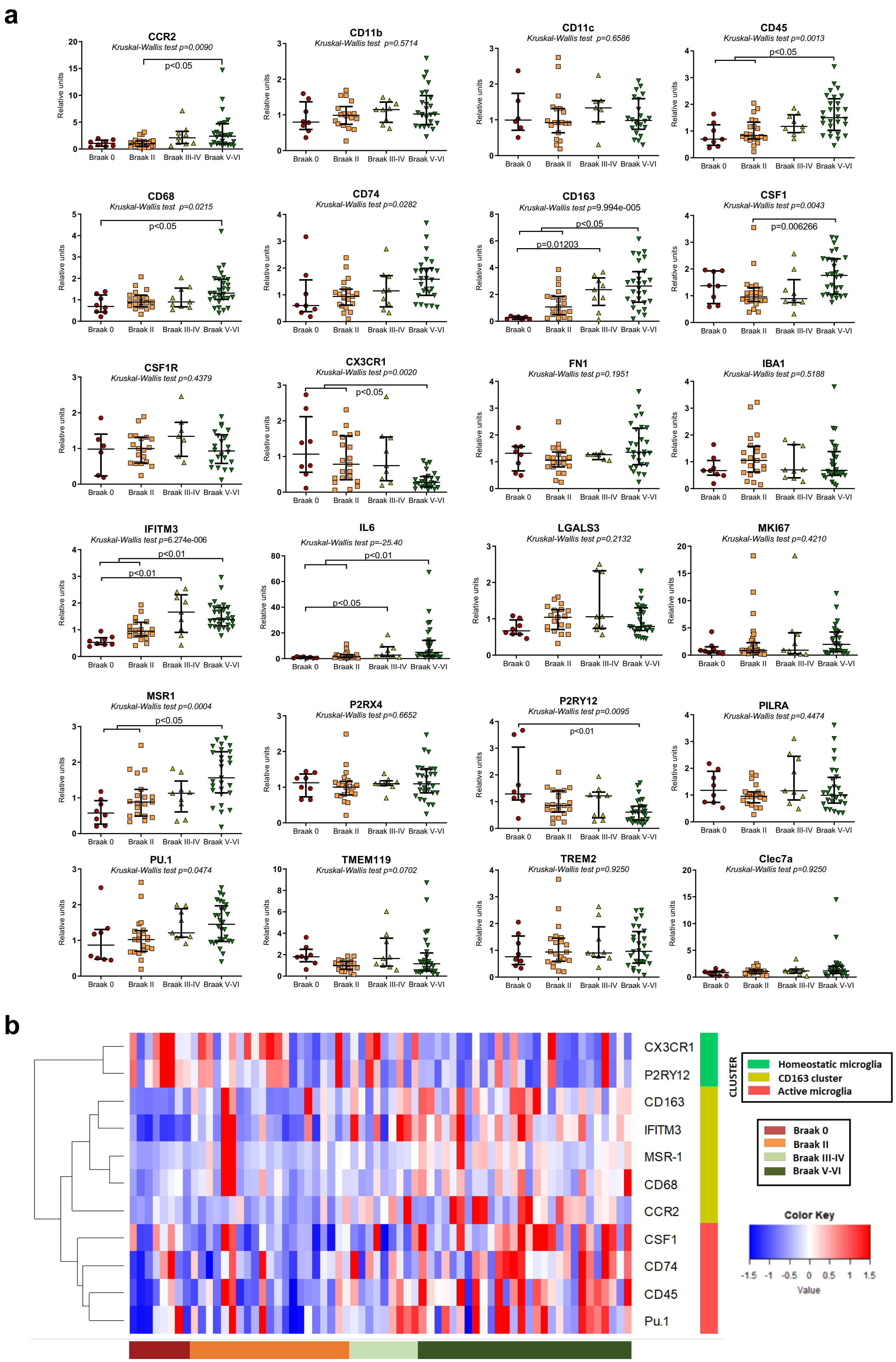
a
CCR2
Kruskal-Wallis test p=0.0090
CD11b
Kruskal-Wallis test p=0.5714
CD11c
Kruskal-Wallis test p=0.6586
CD45
Kruskal-Wallis test p=0.0013
CD68
Kruskal-Wallis test p=0.0215
CD74
Kruskal-Wallis test p=0.0282
CD163
Kruskal-Wallis test p=9.994e-005
CSF1
Kruskal-Wallis test p=0.0043
CSF1R
Kruskal-Wallis test p=0.4379
CX3CR1
Kruskal-Wallis test p=0.0020
FN1
Kruskal-Wallis test p=0.1951
IBA1
Kruskal-Wallis test p=0.5188
IFITM3
Kruskal-Wallis test p=6.274e-006
IL6
Kruskal-Wallis test p=-25.40
LGALS3
Kruskal-Wallis test p=0.2132
MKI67
Kruskal-Wallis test p=0.4210
MSR1
Kruskal-Wallis test p=0.0004
P2RX4
Kruskal-Wallis test p=0.6652
P2RY12
Kruskal-Wallis test p=0.0095
PILRA
Kruskal-Wallis test p=0.4474
PU.1
Kruskal-Wallis test p=0.0474
TMEM119
Kruskal-Wallis test p=0.0702
TREM2
Kruskal-Wallis test p=0.9250
Clec7a
Kruskal-Wallis test p=0.9250
Relative units
Braak 0
Braak II
Braak III-IV
Braak V-VI
b
CX3CR1
P2RY12
CD163
IFITM3
MSR-1
CD68
CCR2
CSF1
CD74
CD45
Pu.1
CLUSTER
Homeostatic microglia
CD163 cluster
Active microglia
Braak 0
Braak II
Braak III-IV
Braak V-VI
Color Key
Value

Figure S1

a **Spearman correlation between TREM2 expression and microglial or CD163 related genes**

		SALL1	MEF2A	CD163	IFITM2	IFITM3	F13A1
**Trem2**	*r*	**0.526**	**0.572**	0.112	0.077	0.050	0.146
	p	**0.010**	**0.004**	0.612	0.737	0.982	0.506
	n	**23**	**23**	23	23	23	23

b

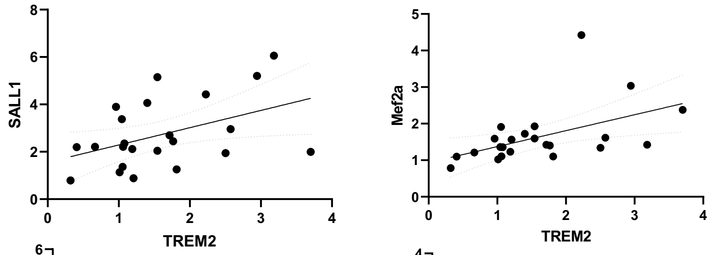

c

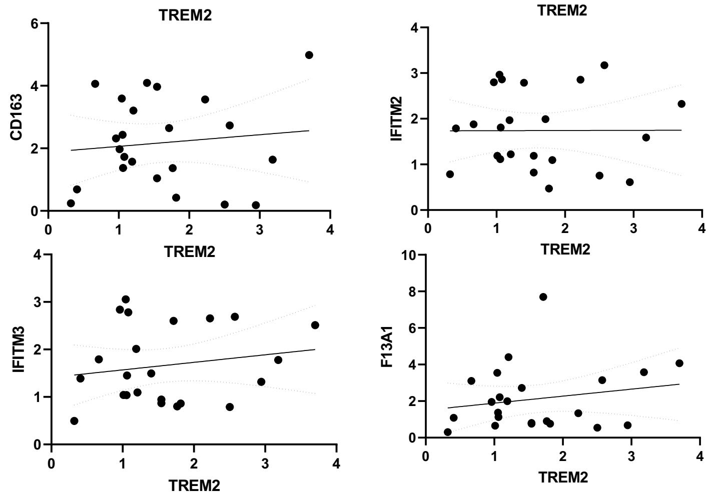

d

		AXL	CST7	MEF2A	CD163	IFITM2	IFIM3	F13A1
**SALL1**	*r*	**0.659**	**0.420**	**0.530**	0.324	0.402	0.410	0.077
	p	**0.010**	**0.040**	**0.009**	0.131	0.081	0.055	0.523
	N	**23**	**23**	**23**	23	23	23	23

Figure S2

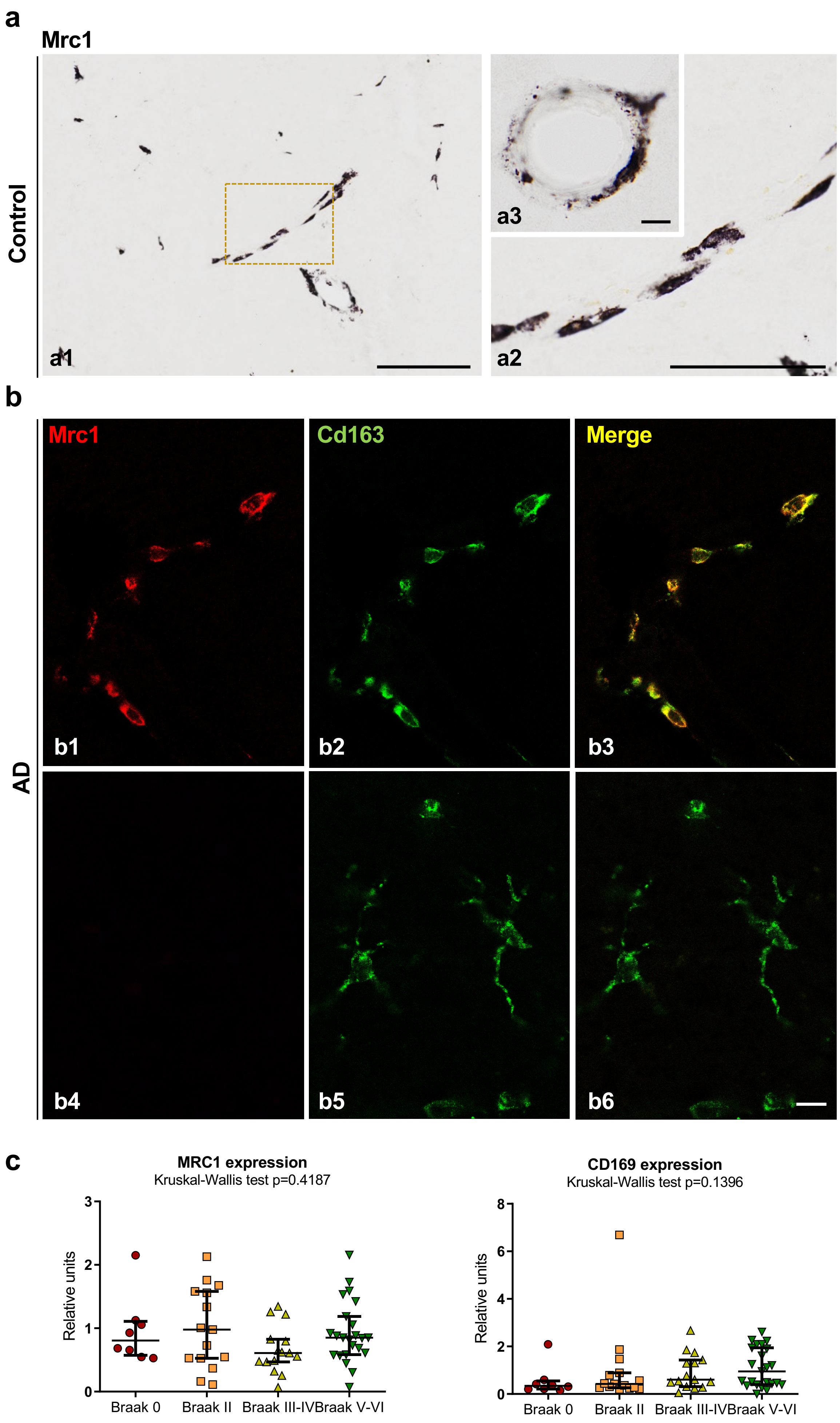
a
Mrc1
Control
a1
a2
a3
b
Mrc1
Cd163
Merge
AD
b1
b2
b3
b4
b5
b6
c
MRC1 expression
Kruskal-Wallis test p=0.4187
Relative units
Braak 0
Braak II
Braak III-IV
Braak V-VI
CD169 expression
Kruskal-Wallis test p=0.1396
Relative units
Braak 0
Braak II
Braak III-IV
Braak V-VI

Figure S3

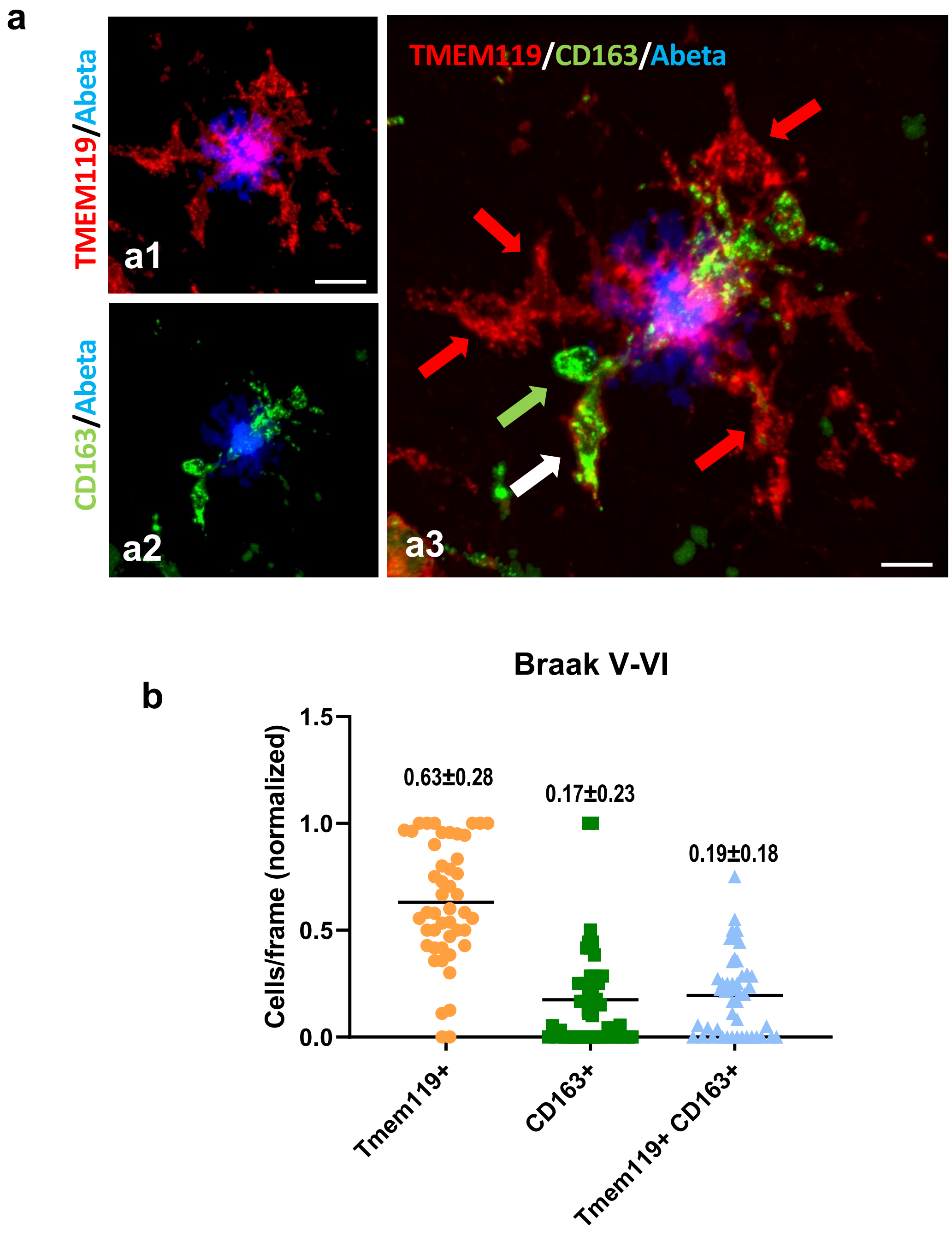
a
TMEM119/Abeta
a1
CD163/Abeta
a2
TMEM119/CD163/Abeta
a3
b
Braak V-VI
Cells/frame (normalized)
1.5
1.0
0.5
0.0
0.63±0.28
0.17±0.23
0.19±0.18
Tmem119+
CD163+
Tmem119+ CD163+

Figure S4

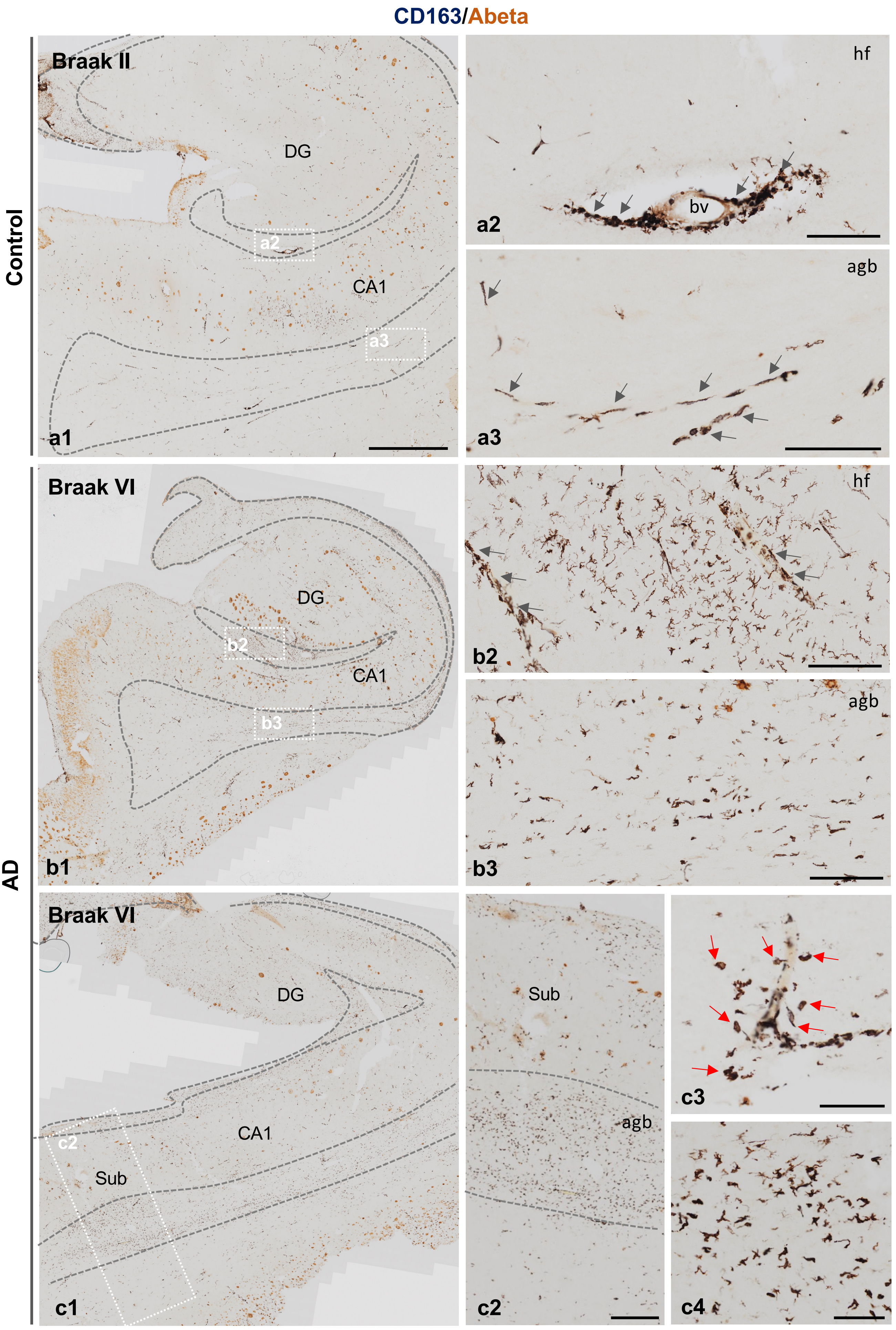
CD163/Abeta
Control
AD
Braak II
DG
CA1
a2
a3
a1
hf
bv
a2
agb
a3
Braak VI
DG
b2
CA1
b3
b1
hf
b2
agb
b3
Braak VI
DG
CA1
c2
Sub
c1
Sub
agb
c2
c3
c4

Figure S5

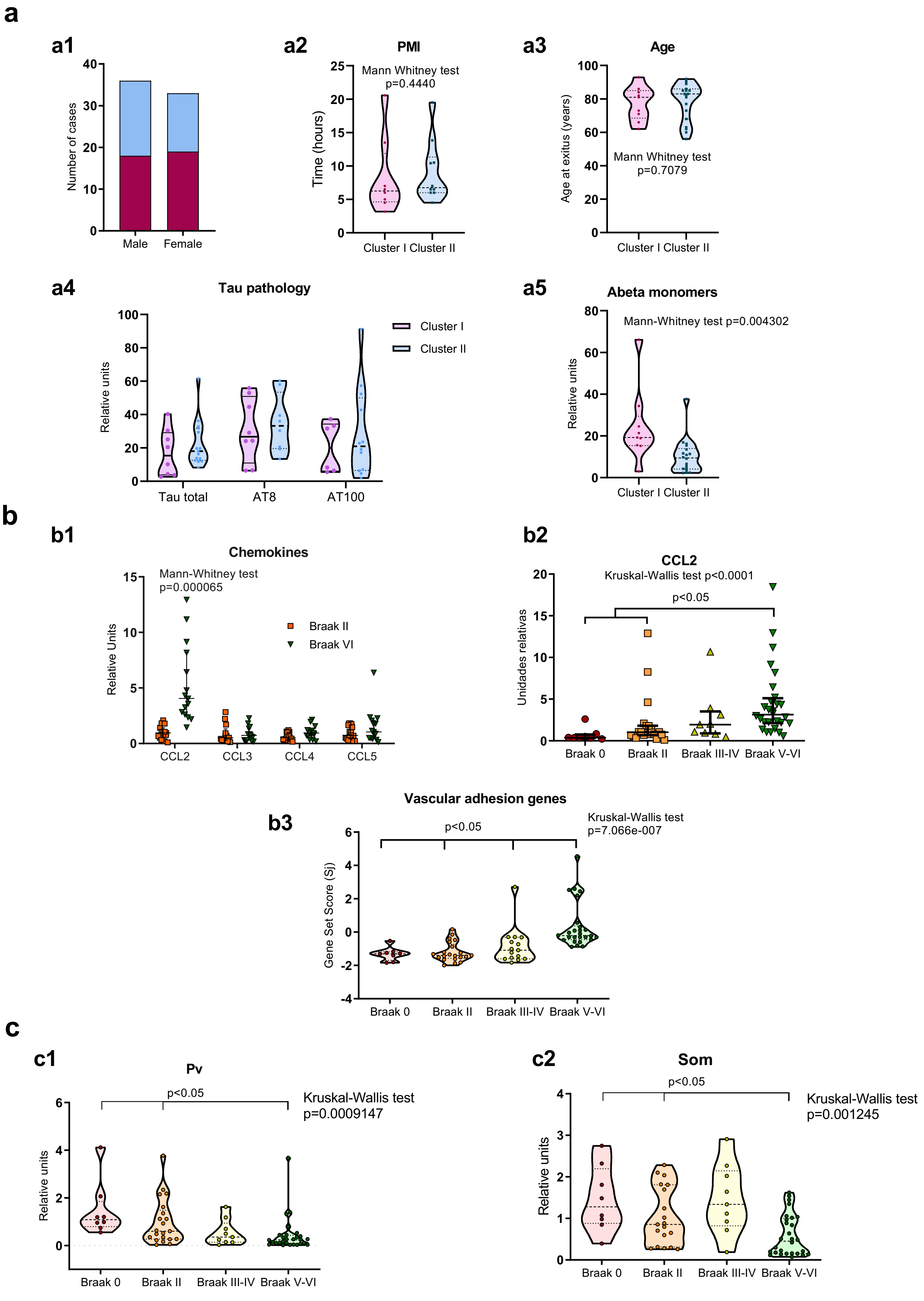
a
a1
Number of cases
Male
Female
a2
PMI
Mann Whitney test
p=0.4440
Time (hours)
Cluster I Cluster II
a3
Age
Age at exitus (years)
Mann Whitney test
p=0.7079
Cluster I Cluster II
a4
Tau pathology
Relative units
Cluster I
Cluster II
Tau total
AT8
AT100
a5
Abeta monomers
Mann-Whitney test p=0.004302
Relative units
Cluster I Cluster II
b
b1
Chemokines
Mann-Whitney test
p=0.000065
Relative Units
Braak II
Braak VI
CCL2
CCL3
CCL4
CCL5
b2
CCL2
Kruskal-Wallis test p<0.0001
p<0.05
Unidades relativas
Braak 0
Braak II
Braak III-IV
Braak V-VI
b3
Vascular adhesion genes
Kruskal-Wallis test
p=7.066e-007
p<0.05
Gene Set Score (Sj)
Braak 0
Braak II
Braak III-IV
Braak V-VI
c
c1
Pv
p<0.05
Kruskal-Wallis test
p=0.0009147
Relative units
Braak 0
Braak II
Braak III-IV
Braak V-VI
c2
Som
p<0.05
Kruskal-Wallis test
p=0.001245
Relative units
Braak 0
Braak II
Braak III-IV
Braak V-VI

Figure S6

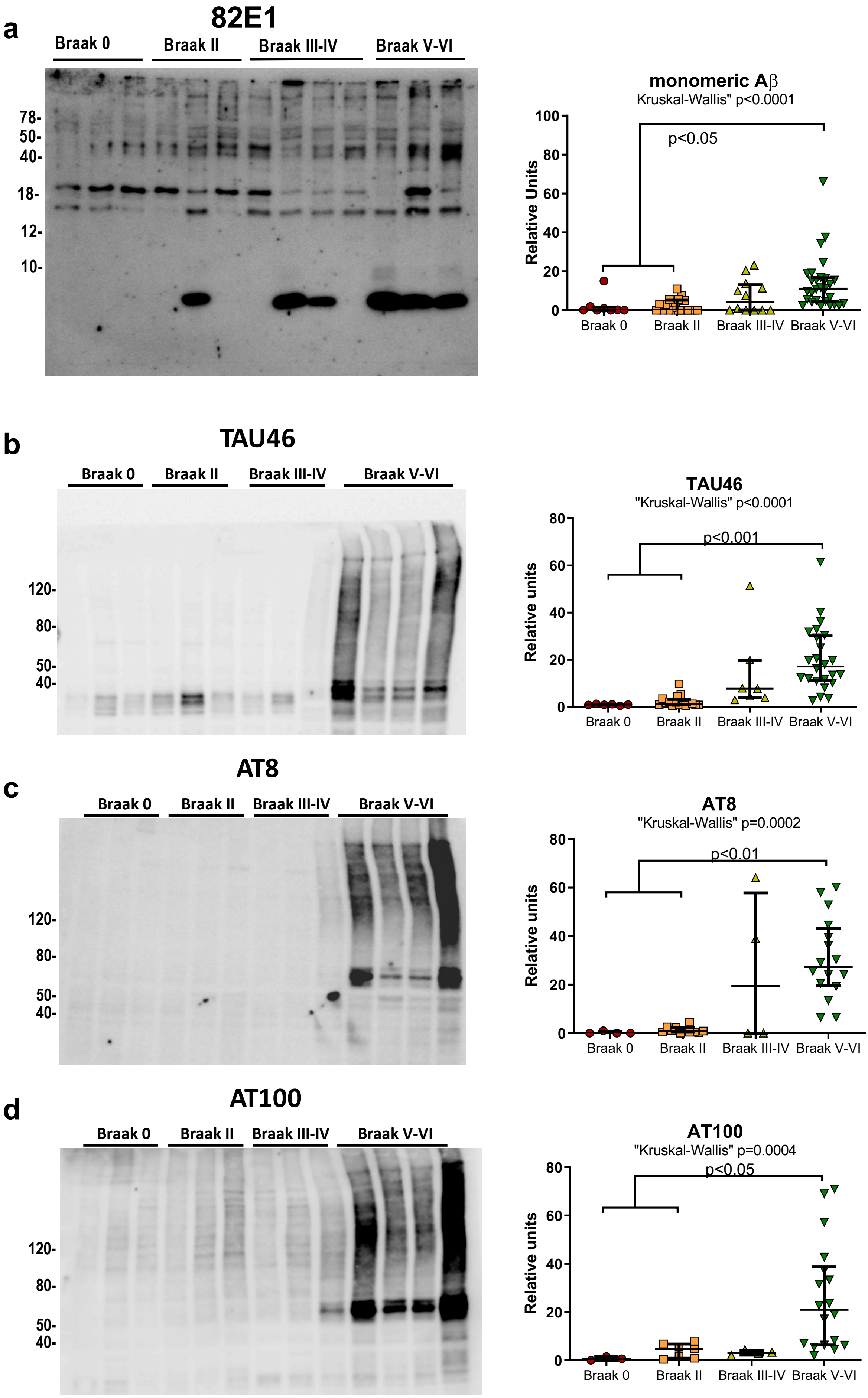
a
82E1
Braak 0
Braak II
Braak III-IV
Braak V-VI
78-
50-
40-
18-
12-
10-
monomeric Aβ
Kruskal-Wallis" p<0.0001
p<0.05
Relative Units
Braak 0
Braak II
Braak III-IV
Braak V-VI
b
TAU46
Braak 0
Braak II
Braak III-IV
Braak V-VI
120-
80-
50-
40-
TAU46
"Kruskal-Wallis" p<0.0001
p<0.001
Relative units
Braak 0
Braak II
Braak III-IV
Braak V-VI
c
AT8
Braak 0
Braak II
Braak III-IV
Braak V-VI
120-
80-
50-
40-
AT8
"Kruskal-Wallis" p=0.0002
p<0.01
Relative units
Braak 0
Braak II
Braak III-IV
Braak V-VI
d
AT100
Braak 0
Braak II
Braak III-IV
Braak V-VI
120-
80-
50-
40-
AT100
"Kruskal-Wallis" p=0.0004
p<0.05
Relative units
Braak 0
Braak II
Braak III-IV
Braak V-VI

Figure S7

Table S1. Taqman probes for RT-qPCR

Probe	Reference
β-actin	Hs99999903_m1
CCL2	Hs00234140_m1
CCR2	Hs00704702_s1
CDH5	Hs00901465_m1
CD11b	Hs00355885_m1
CD11c	Hs00174217_m1
CD163	Hs00174705_m1
CD45	Hs04189704_m1
CD68	Hs02836816_g1
CD74	Hs00269961_m1
CLEC7A	Hs01902549_s1
CSF1	Hs00174164_m1
CSF1R	Hs00911250_m1
CX3CR1	Hs01922583_s1
CYC1	Hs00357717_m1
FN1	Hs00365052_m1
GAPDH	Hs03929097_g1
IBA1	Hs00610419_g1
ICAM2	Hs00609563_m1
IFITM3	Hs03057129_s1
IL6	Hs00985639_m1
LGALS3	Hs00173587_m1
MKI67	Hs01032443_m1
MSR1	Hs00234007_m1
NPY	Hs00173470_m1
PECAM1	Hs01065279_m1
PU.1	Hs02786711_m1
PV	Hs00161045_m1
P2RX4	Hs00602442_m1
P2RY12	Hs01881698_m1
RPL13	Hs00744303_s1
SELE	Hs00174057_m1
SST	Hs00356144_m1
TMEM119	Hs01938722_u1
TREM2	Hs00219132_m1
UBE2D2	Hs00366152_m1
18S	Hs03003631_g1

Table S2: Taqman probes included in the Fluidic Cards.

Array microfluidic cards		
**Cell specificity**	**Probe**	**Reference**
Monocytes / MDC	CD163	Hs00174705_m1
	IFITM2	Hs00829485_sH
	IFITM3	Hs03057129_s1
	F13A1	Hs01114178_m1
	NPC2	Hs00197565_m1
	TAGLN2	Hs00761239_s1
	HLA-dr	Hs04192463_mH
	LILRB2	Hs00275975_m1
	CXCL16	Hs00222859_m1
	NR4A1	Hs00374226_m1
Perivascular Macrophages	MRC-I	Hs00267207_m1
	CD169/Siglec1	Hs00224991_m1
Microglia	Trem2	Hs00219132_m1
	AXL	Hs01064444_m1
	CST-7	Hs00175361_m1
	SALL1	Hs01548765_m1
	MEF2a	Hs01050409_m1
Housekeeping genes	GAPDH	Hs02786624_g1
	18S	Hs03003631_g1
	HPRT1	Hs99999909_m1
	UBC	Hs00824723_m1
